# *Stenotrophomonas maltophilia* complex: insights into evolutionary relationships, global distribution and pathogenicity

**DOI:** 10.3389/fcimb.2023.1325379

**Published:** 2024-01-10

**Authors:** Kun Li, Keyi Yu, Zhenzhou Huang, Xiao Liu, Li Mei, Xiaodong Ren, Xuemei Bai, He Gao, Zhiwen Sun, Xiaoning Liu, Duochun Wang

**Affiliations:** ^1^ School of Public Health, Lanzhou University, Lanzhou, China; ^2^ National Key Laboratory of Intelligent Tracking and Forecasting for Infectious Diseases, National Institute for Communicable Disease Control and Prevention, Chinese Center for Disease Control and Prevention, Beijing, China; ^3^ Microbiology Laboratory, Hangzhou Center for Disease Control and Prevention, Hangzhou, China; ^4^ National Pathogen Resource Center, Chinese Center for Disease Control and Prevention, Beijing, China

**Keywords:** *Stenotrophomonas maltophilia* complex, evolution, global distribution, pathogenicity, swimming motility, biofilm

## Abstract

**Introduction:**

*Stenotrophomonas maltophilia* complex (Smc) comprises opportunistic Gram-negative bacilli responsible for various nosocomial infections. Limited data exists concerning its evolutionary lineage, global prevalence and pathogenicity.

**Methods:**

We conducted an extensive genomic analysis on 734 Smc genomes, of which 90 were newly sequenced and isolated from different patients. The species composition and evolutionary relationships of Smc were examined using core protein sequence analysis. Pathogenicity evaluation was used by assays for swimming motility, biofilm formation and identification of virulence factors. The broth microdilution method was used to evaluate the drug resistance spectrum of clinical isolates.

**Results:**

Phylogenetic analyses delineated 24 species-level clades, dominated by *S. maltophilia* (42.8%), *S. sepilia* (13.6%) and *S. geniculata* (9.9%). Geographically, strains were primarily distributed in Europe (34.2%), Asia (33.7%) and North America (24.0%), with intricate global distribution patterns. Meanwhile, 154 virulence-associated genes and 46 antimicrobial resistance genes within Smc were identified. These genes encoded span various functions, including motility, adherence, toxin, RND antibiotic efflux pumps, beta-lactamases and aminoglycoside-modifying enzymes. Moreover, significant variations were indicated in swimming motility and biofilm-forming capability across the different species, with *S. sepilia* exhibiting superior levels of both traits. Additionally, no statistically significant discrepancy was detected among Smc species to other antibiotics, despite the fact that all *S. geniculata* isolates were resistant to Ceftazidime and much higher than other species.

**Conclusion:**

Our findings indicate the need to pay increased attention to other mainstream species of Smc besides *S. maltophilia* in order to better manage Smc-related infections and tailor effective treatment strategies.

## Introduction

The genus *Stenotrophomonas* comprises aerobic Gram-negative bacteria and belongs to the family *Lysobacteraceae*. It exhibits high diversity and metabolic versatility and can be found ubiquitously in the environment, including water, soil, plant rhizospheres and animals (Patil et al., 2016; Brooke, 2021). Currently, the genus *Stenotrophomonas* consists of 25 validated species, as documented by the List of Prokaryotic Names with Standing in Nomenclature (https://lpsn.dsmz.de/). Among them, *Stenotrophomonas maltophilia* stands as the first identified and clinically most significant species (Palleroni and Bradbury, 1993). Thorough taxonomic and phylogenomic investigations have demonstrated that *S. maltophilia* includes multiple cryptic species, giving rise to the *Stenotrophomonas maltophilia* complex (Smc) and a distinction that eludes conventional classification methods (Ochoa-Sánchez and Vinuesa, 2017; Kumar et al., 2020; Singh et al., 2023). This assemblage involves previously documented taxa, including *Pseudomonas hibiscicola*, *Pseudomonas beteli*, *S. africana*, *S. geniculata*, *S. maltophilia*, *S. pavanii* and *S. sepilia*, with *S. maltophilia* being the prevalent species (Rhee et al., 2013; Kumar et al., 2020; Gautam et al., 2021).

*S. maltophilia* constitutes an emerging opportunistic pathogen that is accountable for a wide range of nosocomial infections, including respiratory infections, bacteremia, skin and soft tissue infections, eye infections and infections connected to medical implants (Brooke, 2021). A pivotal virulence factor contributing to its pathogenicity is its capacity for attachment to both inanimate surfaces and tissues of the host, resulting in the formation of biofilm communities that possess innate resistance to antibiotics and immune responses (Pompilio et al., 2008; Pompilio et al., 2020). Moreover, it has been observed that *S. maltophilia* has the ability to establish colonization in the respiratory tract of individuals with cystic fibrosis, hence elevating the risk of pulmonary exacerbations and mortality (Waters et al., 2011; Waters et al., 2013). Furthermore, the bacterium’s inherent ability to resist widely utilized antimicrobial agents, including cephalosporins, carbapenems, aminoglycosides, and macrolides, stems from low membrane permeability, active antibiotic efflux pumps, and antibiotic-modifying enzymes (Wang et al., 2018; Gil-Gil et al., 2020). This resistance poses a substantial threat to patients receiving treatment, particularly those with compromised immune systems (Zhao et al., 2018; Gil-Gil et al., 2020). Additionally, *S. maltophilia* can play a significant role in microbiological co-infections. For instance, *S. maltophilia* has been found to provide significant levels of protection against antibiotics for *Pseudomonas aeruginosa*, which would otherwise be susceptible to them (Bottery et al., 2022; Lee et al., 2022) and interfere with the isolation cultures and drug sensitivity tests of *Mycobacterium tuberculosis*, potentially leading to alterations in treatment plans and an escalation in the burden of the disease (Li et al., 2023).

Prior research provides evidence for the presence of various species inside the Smc that are connected with human infections (Patil et al., 2018; Gautam et al., 2021). However, there are large knowledge gaps in our understanding of clinically and environmentally relevant aspects of these closely related species, such as the prevalence or proportion, the virulence factors along with the antimicrobial-resistance characteristics of each species of Smc. The wealth of genome sequences available in public databases offers a valuable resource to bridge these knowledge gaps.

In this study, we analyzed 90 newly sequenced clinical isolates from diverse regions of China together with genomes with strict quality control from public databases. We reconstructed the phylogenetic relationship of the genus *Stenotrophomonas* and updated the species composition of Smc. Furthermore, we explore the global population distribution structure of these species, investigate the sources of isolates, and analyze the presence of virulence and resistance genes. Additionally, the differences in biofilm formation ability, swimming ability and drug resistance phenotype among the 90 clinically derived Smc that we have newly sequenced were further evaluated. This study enhances the comprehension of the population structure and worldwide distribution of different species within the Smc, and provides the basis for gaining further insights into the species-specific clinical characteristics, potential pathogenicity, and resistance spectrum of the Smc.

## Materials and methods

### Bacterial strains and DNA isolation

All 90 strains in this study were routinely isolated from clinical specimens of different patients in China between 2019 and 2023 (**Supplementary Table S1**). The source from which most isolates were obtained was human sputum, followed by trachea tube, suggesting that most strains isolate from respiratory. All strains were identified as *S. maltophilia* by MALDI-TOF MS and DNA was extracted using the Genomic DNA Extraction Kit (Tiangen biochemical technology Co. Ltd., Beijing, China).

### Whole-genome data collection and sequencing

A total of 758 *S. maltophilia* genomes were analyzed in this study. Among them, 90 clinical strains were newly sequenced (**Supplementary Table S1**). Another 668 uncontaminated genomes labeled “*Stenotrophomonas maltophilia*” with less than 200 contigs were screened and included from NCBI (https://www.ncbi.nlm.nih.gov/datasets/genome/) as of March 2023 (**Supplementary Table S2**).

Genomes were sequenced using the Illumina HiSeq 2000 platform. The raw sequence data was evaluated by FastQC (http://www.bioinformatics.babraham.ac.uk/projects/fastqc/), and the low-quality reads were filtered. The reads data was assembled into contigs using SOAP *de novo* v2.04 (Li et al., 2019). QUAST v5.0.2 (Gurevich et al., 2013) software was applied to evaluate the genome assembly. The genome size, number of coding sequences (CDSs), and GC content were estimated using Prokka v1.13.3 (Seemann, 2014).

### Calculation of average nucleotide identity and phylogenetic tree construction

FastANI (Jain et al., 2018) was employed to calculate the pairwise average nucleotide identity (ANI). The similarity matrix was imported into R and plotted as a heatmap of all strains. The strains belonged to the same species when the ANI value was above 95%. The genomes that could not be assigned to a known species were compared against all type strain genomes available in the TYGS (Type (Strain) Genome Server, https://tygs.dsmz.de/) database via the MASH algorithm to determine closely related type strains.

Phylogenetic analysis of the genus *Stenotrophomonas* follows the previously described method (Huang et al., 2022). Ten representative genomes were selected to cover the overall genetic distance of each species for those species with more than 10 available sequenced genomes, and all the genomes for the species with less than 10 publicly available sequences were adopted. Finally, a total of 190 strains were included, and the non-redundant homologous gene set was computed by CD-HIT (Fu et al., 2012). Next, BLASTp was used to search the homologous proteins in the nonredundant homologous protein set of each strain with a cutoff value of ≥ 90% sequence identity and ≥ 60% length coverage. Each single-copy core protein sequence was aligned separately using ClustalW2 and then merged. Iqtree v1.6.11 (Minh et al., 2020) was used to construct the phylogenetic trees by the maximum-likelihood method based on all these core SNPs (bootstrap replications, 1000). The visualization and beautification of the phylogenetic tree were accomplished by the tvBOT online tool (https://www.chiplot.online/tvbot.html) (Xie et al., 2023).

### Identification of virulence-associated genes and antibiotic resistance genes

Nucleotide sequences predicted by Prokka v1.13.3 (Seemann, 2014) and produce *.gff output files were used for the following identification of core genes by Roray v2.3.12 (Page et al., 2015), with default parameters. To identify the virulence-associated genes, the protein sequences of all genomes were aligned using BLASTp with a relatively relaxed cutoff (60% identity, 60% length coverage) and an E value of 1e-5 against the data set from the VFDB (Liu et al., 2022). The antibiotic resistance genes (ARGs) were predicted using Resistance Gene Identifier (RGI) v6.0.2 with cutoffs of 75% identity and 80% length coverage against the data set from the Comprehensive Antibiotic Resistance Database (CARD, https://card.mcmaster.ca/) (Alcock et al., 2023).

### Swimming motility assay

Logarithmically grown bacterial culture was adjusted to an optical density at 600 nm (OD_600_) of 0.4–0.5, and an aliquot (2 μL) of the culture was transferred into the LB semisoft (0.15% agar) agar motility plates, followed by incubation at 37°C for 22–24 h. Three parallel controls were set up, and the bacterial expansion zone diameters (millimeters) were measured.

### Biofilm formation assay

The crystal violet (CV) staining method was used to quantify the ability of each isolate to form biofilm as previously described (Isom et al., 2022). Bacterial cultures of strains were grown overnight in LB medium, and 2 mL was next inoculated into an individual test sterile polystyrene tube (Falcon, Coning; NY, USA) at a 1:50 dilution. Incubation was at 37 °C for 24 h without shaking, under an aerobic atmosphere, and the OD_600_ of each bacterial culture was measured. Then, non-adherent bacteria were removed by washing three times with sterile water carefully, and biofilm samples were fixed for 30 min at 60°C, dyed for 30 min with 0.1% CV, followed by careful rinsing with water to remove excess dyes, and solubilization of the crystal violet using dimethylsulfoxide (DMSO) for 1 h. Finally, the OD_570_ was measured, and the OD_570_/OD_600_ ratio was used to represent the biofilm formation ability.

### Antibiotic susceptibility test

A total of 90 Smc isolates were tested for antimicrobial susceptibility. Minimum inhibitory concentrations (MICs) of 15 antimicrobial agents (Levofloxacin (LVX), Trimethoprim/Sulfamethoxazole (SXT), Minocycline (MH), Ceftazidime (CAZ), Amikacin (AN), Ampicillin/Sulbactam (SAM), Aztreonam (ATM), Cefepime (FEP), Ceftriaxone (CRO), Ciprofloxacin (CIP), Gentamicin (GM), Imipenem (IPM), Ertapenem (ETP), Piperacillin/Tazobactam (TZP), and Tobramycin (NN)) were detected by the broth microdilution method, and the resistance was defined using the Clinical and Laboratory Standards Institute (CLSI) criteria (Clinical Laboratory Standards Institute, 2023). *Pseudomonas aeruginosa* ATCC 27853 and *Escherichia coli* ATCC 25922 were used as control strains.

### Statistical analysis

SPSS 26.0 (IBM Corp., Armonk, NY, USA) was used to conduct the statistical analysis. For categorical variables, the χ^2^ test or Fisher’s exact test was used to determine statistical significance. P < 0.01 was considered to indicate statistical significance.

### Data availability

The genome sequences of the 90 clinical Smc strains sequenced in this study have been deposited at GenBank/DDBJ/ENA under the BioProject ID no. PRJNA1019301.

## Results

### Phylogenetic reconstruction of Smc and its composition of species

**Figure 1:**
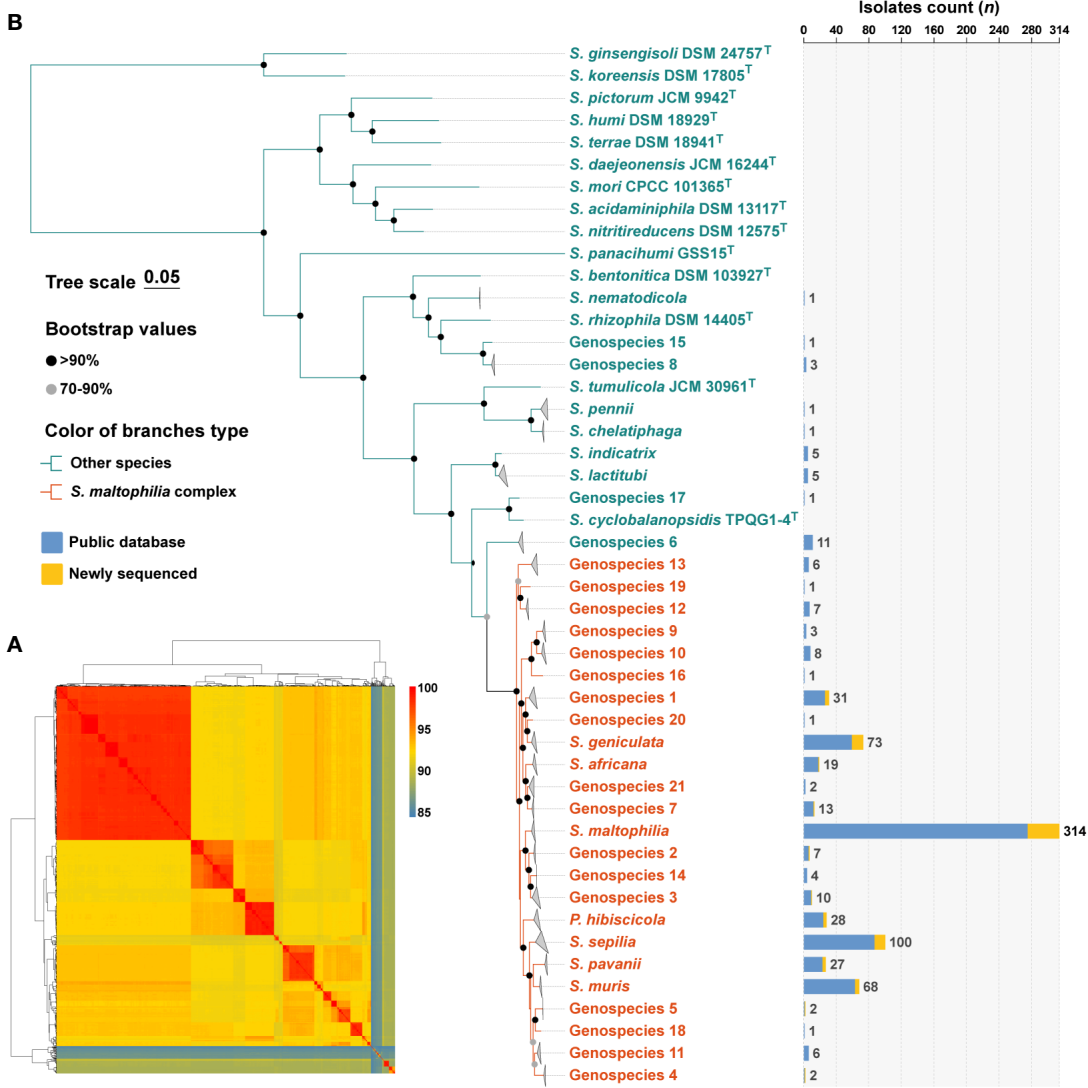
Species identification of 758 *S. maltophilia* genomes and phylogenetic analysis of the genus *Stenotrophomonas*. **(A)** The heatmap shows that pairwise average nucleotide identity of 758 genomes of *S. maltophilia* and type strains of the genus *Stenotrophomonas* and *P. hibiscicola*. **(B)** Phylogenetic placement of *S. maltophilia* complex strains within the genus *Stenotrophomonas*. Genomes belonging to the same species were folded and represented by triangles. Orange was used to indicate the evolutionary branches that make up the Smc. The robustness of tree topology was evaluated with 1,000 bootstrap replications, and values >70% are shown at the nodes of the branches. The stacked column chart represents the number of each species collected in this study, with colors indicating whether they were from public databases or newly sequenced.

The 758 genomes were compared with type strains of the *Stenotrophomonas* genus by ANI calculations, and a total of 32 species-level clades were identified (**Figure 1A**). Among them, in addition to the 637 genomes assigned to 11 well-defined species, 121 genomes remained unassigned to any known species. Subsequently, the 121 strains were compared to the TYGS database, resulting in their identification as *Stenotrophomonas* spp., and could be further assigned to 21 novel *Stenotrophomonas* species named Genospecies 1 through Genospecies 21 (**Supplementary Table S2**). It is worth noting that out of the 734 strains classified as “*S. maltophilia*”, only 314 (41.4%) were confirmed to be *S. maltophilia*. The majority of the remaining strains belonged to different species within the genus *Stenotrophomonas*.

The determination of phylogenetic connections among *Stenotrophomonas* spp. was based on the analysis of core protein sequences (**Figure 1B**), and the resulting phylogenetic tree revealed 24 species-level clades of Smc. The branches of each species of Smc are characterized by their short and compact arrangement, indicating a close relationship. Consequently, a total of 734 Smc genomes were incorporated into the subsequent analysis, the most prevalent species were *S. maltophilia* (*n* = 314, 42.8%), *S. sepilia* (*n* = 100, 13.6%), *S. geniculata* (*n* = 73, 9.9%), *S. muris* (*n* = 68, 9.3%), and Genospecies 1 (*n* = 31, 4.2%).

### Population structure and global distribution of Smc

**Figure 2:**
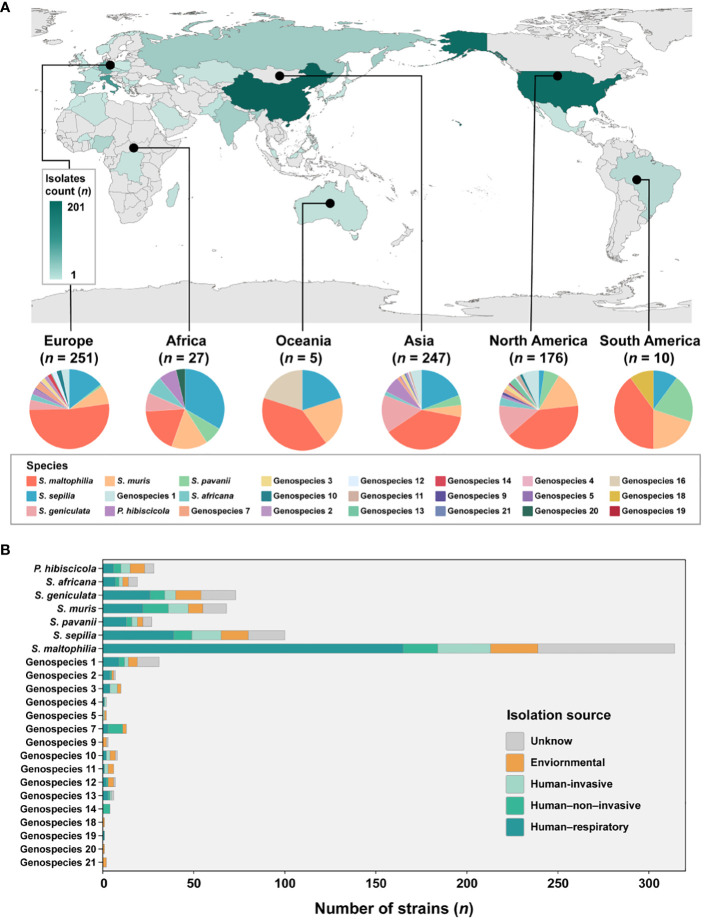
Global distribution of species and their composition by isolation source in Smc. **(A)** Geographic distribution of the 734 *S. maltophilia* complex strains. The map is color-coded by country to indicate the number of strains. **(B)** Barplot shows the number of strains per species colored by isolation source for the entire strain collection.

The analysis incorporated 734 strains from 39 different countries across 6 continents, with the exclusion of 16 isolates lacking information regarding their geographic origin (**Figure 2A**). These strains were predominantly obtained from China (*n* = 201, 27.4%), the United States (*n* = 174, 23.7%), Italy (*n* = 90, 12.3%), Germany (*n* = 60, 8.2%), and Spain (*n* = 28, 3.8%). The majority of strains are geographically spread over Europe (*n* = 251, 34.2%), Asia (*n* = 247, 33.7%) and North America (*n* = 176, 24.0%). Subsequently, smaller proportions of strains are found in Africa (*n* = 27, 3.6%), South America (*n* = 10, 1.3%) and Oceania (*n* = 5, 0.6%) is the least.

Given the disparity in the distribution of strains between continents, our study concentrated specifically on Europe, Asia and North America. These three continents were chosen due to their more abundant number of strains and species composition. In Europe, the prevalence of *S. maltophilia* was significantly higher (51.8%, *P* < 0.005) compared to the relatively lower occurrence of *S. geniculata* (4.0%, *P* < 0.005). *S. muris* strains in North America were significantly greater at 14.8% (*P* < 0.01). However, *S. sepilia* strains in North America were rather low, accounting for only 2.3% (*P* < 0.001). In contrast, higher proportions of these strains were found on the other two continents (Asia (19.0%), Europe (14.3%)). Additionally, *S. maltophilia* exhibits dominance as the prevailing species in South America and Oceania, while *S. sepilia* strains account for the highest proportion in Africa.

In terms of sample sources of Smc, a majority of the strains (*n* = 471, 64.2%) were obtained from clinical sources. The isolation source was categorized into five distinct groups (**Figure 2B**). The predominant isolates from the human respiratory tract were strains of Genospecies 19 (100%), Genospecies 2 (57.1%) and *S. maltophilia* (52.5%). Genospecies 4 (50.0%), Genospecies 5 (50.0%) and Genospecies 3 (40.0%) contained significantly more human-invasive strains compared with all other isolation sources. The prevalence of human-non-invasive origin strains was found to be significantly higher in Genospecies 14 (100%), Genospecies 7 (61.5%) and *S. muris* (20.6%). Additionally, environmental strains were more common in Genospecies 18 (100%), Genospecies 20 (100%), Genospecies 21 (100%) and Genospecies 9 (66.7%).

### Distribution pattern of virulence-associated genes and ARGs in Smc

**Figure 3:**
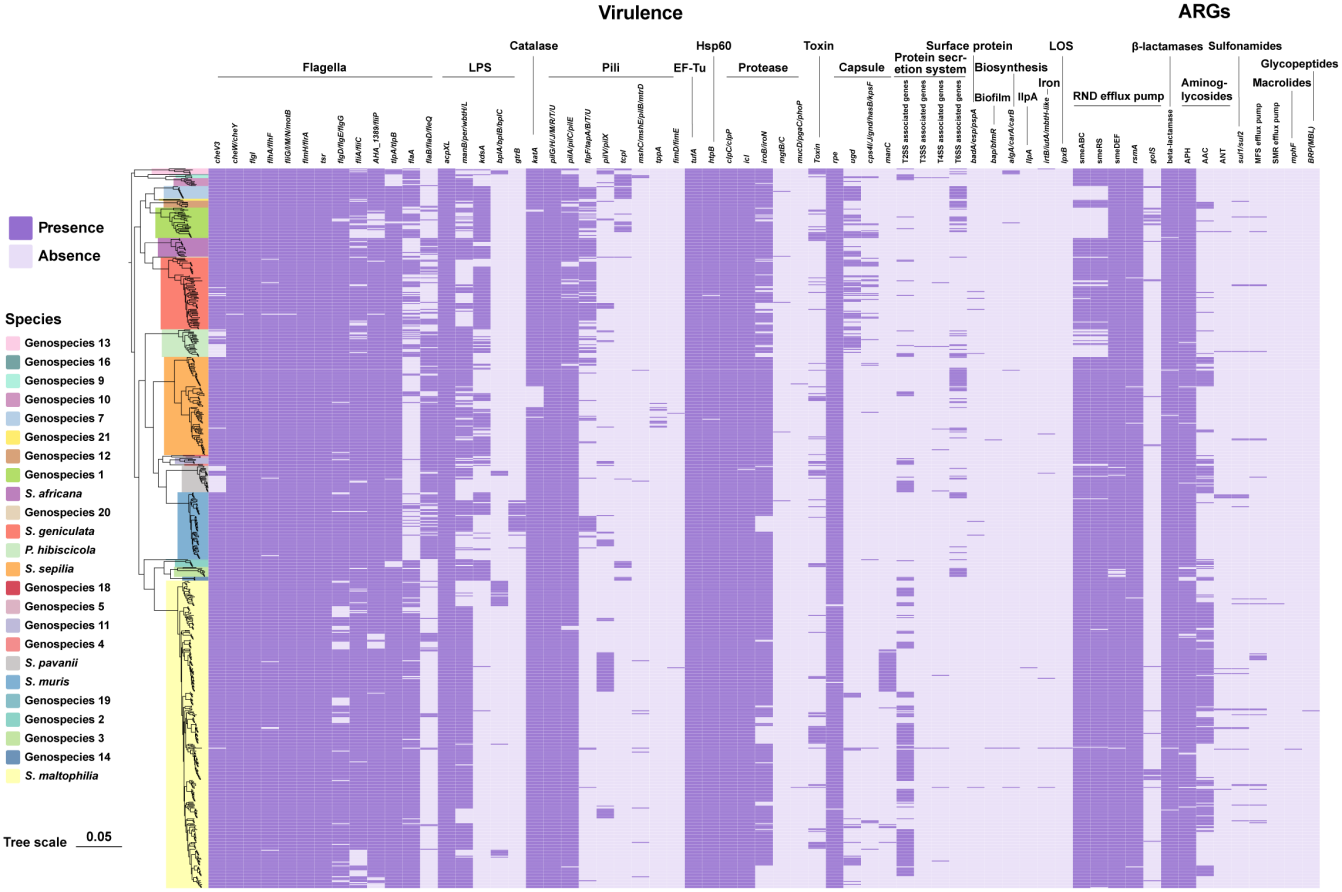
Phylogenomic tree of 734 genomes of Smc and distribution of virulence-associated genes and ARGs. Phylogenetic tree performed by the ML method based on core gene sequences of Smc. The pattern of gene presence or absence is displayed in columns next to the tree. Genes that express comparable functions or those from the same class are integrated.

According to the annotations and predictions made by the VFDB database, 17 virulence factor classes, involving 154 virulence-associated genes, were obtained from 734 Smc genomes (**Table 1**; **Figure 3**), each genome contains an average of 34 virulence genes. Belonging to 7 virulence factor classes (flagella, pili, LPS, capsule, protease, Hsp60 and EF-Tu) including 25 genes were present in almost all of the Smc genomes. In addition, there are variations in the distribution of certain genes among the species of the Smc. Specifically, the *cheA* and *manC* genes were exclusively found in *S. maltophilia*, where they play a crucial role in flagella formation and adhesion, respectively. Moreover, we found genes associated with type VI secretion system (T6SS) in numerous species of the complex, including Genospecies 7 (100%), Genospecies 12 (100%), Genospecies 3 (90%), *P. hibiscicola* (35.7%), Genospecies 1 (35.5%) and *S. sepilia* (33.0%). The presence of the *gtrB* gene, which plays a role in the modification of lipopolysaccharide (LPS) O-antigen, was merely seen in *S. muris*. The *tppA* gene, encoding Type IV pili, was found solely in *S. sepilia*. Similarly, the *xcpS* gene, encoding the general secretion pathway protein F, was only found in Genospecies 11. It is worth noting that a higher percentage of *S. sepilia* strains (22%) lack the *katA* gene, which is a catalase enzyme that plays a role in increasing persistence levels in response to hydrogen peroxide disinfectants and is found in nearly all other genomes.

**Table 1:** The classification of virulence related factors of Smc.

VF-class	Virulence genes	Productions	Functions
Flagella	*cheA, cheB, cheD, cheR, cheV, cheV3, cheW, cheY, fimD, fimE, flaA, flaB, flaD, fleQ, flgD, flgE, flgG, flgI, flhA, flhF, fliA, fliC, fliG, fliI, fliM, fliN, fliP, flmH, flrA, motB, tlpA, tlpB, tsr*	Chemotaxis protein, chemotaxis methyltransferase, flagellin, transcriptional regulator, flagellar protein, flagellar biosynthesis sigma factor, flagellar protein, type I fimbriae, flagellum-specific ATP synthase, short chain dehydrogenase, FleQ protein, membrane-bound chemoreceptor sensing pH and autoinducer-2	Motility, adherence to surfaces, colonization or invasion, maintenance at the infection site, damage to host
Pili	*flpF, mshC, mshE, pilB, mtrD, pilA, pilC, pilE, pilG, pilH, pilJ, pilM, pilR, pilT, pilU, pilV, pilX, tapA, tapB, tapT, tapU, tcpI, tppA,tppF*	Traffic ATPase, MSHA pili minor prepilin protein, MSHA biogenesis protein, trifunctional thioredoxin, resistance system protein, type 4 fimbrial precursor, type IV pilus major pilin, twitching motility protein, twitching ATPase, negative regulator of the major pilin, type IV pilus pseudopilin	Motility, adherence to surfaces, adaptation, nutrient acquisition, biofilm formation, sense initial contact with surfaces, damage to host
T2SS	*exeG, lspF, lspG, xcpR, xcpS, xcpT*	General secretion pathway protein G, general secretion pathway protein F, general secretion pathway protein E	Damage to host, route of protein export across the cytoplasmic membrane,
T3SS	*bscN, invG, spaQ, yopO/ypkA*	ATP synthase, secretin, minor export apparatus protein, effector YopO	Transport virulence factor, protein secretion,
T4SS	*CBU_1566, CBU_1594, lpg2936, ptlH, virB10, virB11, virB4, virB9*	Translocated effector, protein ATPase, channel protein	Transfer of information between cells, Spread of bacterial resistance
T6SS	*clpV1, hcp1, hsiB1/vipA/mglA, hsiC1/vipB/mglB, icmF1/tssM1, rhs/PAAR, tagT, tli1, vasH/clpV, vgrG1b*	AAA+ family ATPase, HcpA-like protein, tubule-forming protein, PAAR family protein, protein TagT, immunity protein, transcriptional regulator,	Effector protein transfer, protein secretion, virulence, chaperone
Toxin	*chuU, cylB, ddhA, dhbC, dhbE, entE, entF, fdeC, fepA, fepB, hlyB, rtxA, rtxB, rtxE, sodB*	Heme permease protein, ABC-type transporter, structural toxin protein, RTX toxin transporter, superoxide dismutase, hemolysin B, adhesin FdeC, enterobactin synthase, isochorismate synthase,	Bacillibactin, enterobactin, adherence, damage to host, proyection aganist hosts
LPS	*bplA, bplB, bplC, gmd, gtrB, kdsA, manB, per, wbtH, wbtL*	Oxidoreductase, acetyltransferase lipopolysaccharide biosynthesis protein, GDP-mannose 4,6-dehydratase, bactoprenol glucosyl transferase, 2-dehydro-3-deoxyphosphooctonate aldolase, phosphomannomutase perosamine synthetase, asparagine synthase, glucose-1-phosphate thymidylyltransferase	Adherence, immune evasion, endotoxin, Toll-like receptor
Anti-resistance	*acrB, adeG*	Acriflavine resistance protein B, cation/multidrug efflux pump	Anti-resistance
Biosynthesis	*carA, carB*	Carbamoyl phosphate synthase	Pyrimidine biosynthesis
Capsule	*cps4I, cpsJ, gmd, gnd, hasB, kpsF, manC, rpe, ugd*	capsular polysaccharide biosynthesis protein, ATP-binding protein, phosphogluconate dehydrogenase, pyrophosphorylase, UDP-glucose dehydrogenase	Adherence
Catalase	*katA*	Catalase	Protection against host defenses
IlpA	*IlpA*	Immunogenic lipoprotein A	Endotoxin
Iron uptake	*irtB, iutA*, *mbtH-like*	Iron-regulated transporter IrtB, ferripyoverdine receptor FpvA, ferric aerobactin receptor IutA	Transcription activation, signaling receptor, transport
LOS	*kdsA, lpxB*	2-dehydro-3-deoxyphosphooctonate aldolase, lipid-A-disaccharide synthase	Endotoxin
Protease	*iroN, clpC, clpP, icl, iroB, mucD, pgaC, phoP, mgtB, mgtC*	ATP-dependent Clp protease proteolytic, isocitratase, glucosyltransferase, serine protease	Adhesion and invasion, fibrinogen cleavage
Hsp60	*htpB*	60K heat shock protein HtpB	Mediate complement-independent attachment to the host cells
Biofilm	*Bap, bfmR*	Biofilm-associated protein, biofilm-controlling response regulator	Biofilm formation
EF-Tu	*tufA*	Elongation factor Tu	Protein synthesis

A total of 46 ARGs, referring to 7 antimicrobial classes of drugs, were identified against the CARD (**Figure 3**). Almost all genomes carry genes encoding resistance-nodulation-cell division (RND) antibiotic efflux pump, beta-lactamase and aminoglycoside phosphotransferase (APH), indicating the principal resistance mechanism of Smc. The prevalence of genes producing aminoglycoside acetyltransferase (AAC) was found to be approximately 37.1%. These genes were predominantly observed in strains of *S. maltophilia*, *S. pavanii*, and *S. speilia.* Another aminoglycoside-modifying enzyme, aminoglycoside nucleotidyltransferase (ANT), was exclusively encoded in *S. maltophilia*, *S. muris*, Genospecies 1 and *P. hibiscicola*. In addition, all strains of *P. hibiscicola*, Genospecies 1, Genospecies 4, and Genospecies 7 did not encode smeABC (a multidrug resistance RND-type efflux pump that confers resistance to aminoglycosides, beta-lactams, and fluoroquinolones) or smeRS (a two-component regulatory system for smeABC). The sulfonamide-resistance-conferring *sul1* or *sul2* were found in 26 strains (3.6%), mostly occurring in clinical strains, which suggests a relatively low prevalence of trimethoprim/sulfamethoxazole-resistant strains in our collection, which is the recommended first-line agent for the treatment of *S. maltophilia* infection.

### Swimming motility and biofilm formation ability

**Figure 4:**
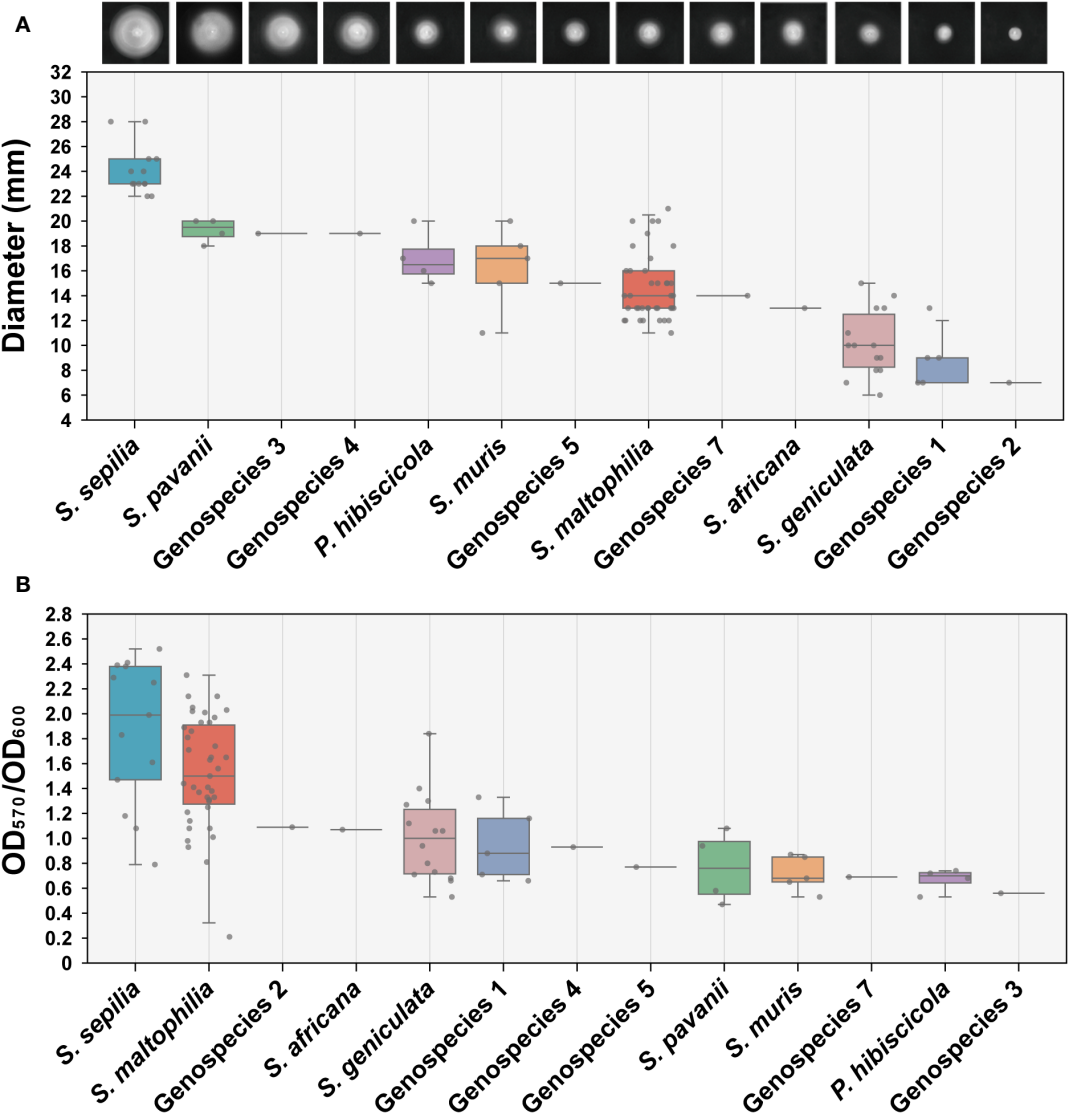
Determination of swimming motility and biofilm formation ability. **(A)** The swimming motility on LB (0.15% agar) measurement results of 90 clinical strains. Each image presented in the study depicts the expansion zone diameters of a single strain that is most representative of the average for its respective species. **(B)** Biofilm formation ability assay (crystal violet staining). Box plot shows the measurement results of each strain.

We conducted an assessment of the swimming motility and biofilm forming capabilities of 90 clinical strains obtained from Smc, encompassing 13 distinct species. The findings indicated significant variations in swimming motility across the different species. *S. sepilia* exhibits the highest level of swimming proficiency, followed by *S. pavanii*, whereas Genospecies 2 displays the lowest swimming motility (**Figure 4A**). Meanwhile, these species have been identified with varying degrees of biofilm-forming capability. *S. maltophilia* and *S. sepilia* in particular have shown stronger biofilm formation activity, followed by Genospecies 2 and *S. africana*, whereas Genospecies 3 was classified as the weakest biofilm producer (**Figure 4B**).

### Antibiotic resistance phenotypic of Smc

A resistance phenotypic test was performed on 15 antibiotics against Smc (**Supplementary Table S3**). Our analyses focused on the resistance of four therapeutic drugs against *S. maltophilia* infections (**Table 2**). From all of the isolates, 44 were susceptible to the four antibiotics tested, whereas three trains, SMYL 1 (*S. geniculata*), SMYL 8 (Genospecies 1), SMYL 55 (*S. maltophilia*), were resistant or intermediary to three antibiotics. Surprisingly, all 14 strains of *S. geniculata* revealed resistance to Ceftazidime and exhibited significantly higher than other species (*P* < 0.001, Fisher’s exact test for *n* < 5). No statistically significant variation was observed between species in terms of resistance to the remaining three antibiotics.

**Table 2:** Four clinical antibiotic susceptibilities of 90 Smc isolates.

Antibiotic*^a^*	MIC (µg/mL) Range	Susceptibility (%)			Non-susceptible species*^b^* (*n*)
		S	I	R	
CAZ	≤ 1 to ≥ 32	50 (55.6)	7 (7.7)	33(36.7)	*S. mal* (17), *S. gen* (14), *S. sep* (2), G1 (2), *S. afr* (1), *S. muris* (1), G 2 (1), G 3 (1), G 7 (1)
LEV	≤ 1 to ≥ 8	79 (87.8)	5 (5.5)	6 (6.7)	*S. mal* (3), *S. gen* (2), *S. mur* (2) *S. sep* (2), *S. pav* (1), *P. hib* (1), G 1 (1)
MH	≤ 1 to 8	87 (96.7)	3 (3.3)	0	*S. mal* (1), *S. gen* (1), G 1 (1)
SXT	≤ 1/19 to ≥ 4/76	88 (97.8)	–	2 (2.2)	*S. mal* (1), *S. mur* (1)

^a^Antibiotic: CAZ, Ceftazidime; LVX, Levofloxacin; SXT, Trimethoprim/Sulfamethoxazole; MH, Minocycline.

^b^Species: *S. gen*, *S. geniculata*; *S. mal*, *S. maltophilia*; *S. sep*, *S. sepilia*; *S. afr*, *S. aficana*; *S. mur*, *S. muris*; *S. pav*, *S.pavanii*; *P. hib*, *P. hibiscicola*; G 1, Genospecies 1; G 2, Genospecies 2; G 3, Genospecies 3; G 7, Genospecies 7.

S, susceptible; R, resistance; I, intermediate.

## Discussion

In this study, we constructed an extensive genome dataset containing 734 strains by combining 90 newly sequenced genomes of Smc. The results of phylogenetic analysis revealed that 24 species consist of Smc, and the topological structure coincided with previous researches (Patil et al., 2018; Gröschel et al., 2020). We incorporate the introduction of *S. muris*, a recently described species, into the species composition of the Smc. Based on the number of strains, *S. maltophilia* remained the dominant species within Smc (42.8%). Moreover, it is noteworthy that *S. sepilia*, *S. geniculata*, and *S. muris* have emerged as the dominant emerging strains in the Smc. Importantly, little is known about the pathogenicity of these species in humans, warranting further investigation.

Matthias I. Gröschel et al. subdivided Smc into 23 monophyletic lineages and clarified the global prevalence of particular lineages (Gröschel et al., 2020). In this study, significant differences have been observed in Smc population structures across continents. The species of Smc in Asia, Europe, and North America are rich, with *S. maltophilia* overwhelmingly dominant. The species composition of Smc in Africa, Oceania and South America is more homogeneous. *S. sepilia* was the mainstream species in Africa and had a higher prevalence compared to the other continents. The complex distribution pattern seen in this study further substantiates the notion of a protracted evolutionary trajectory for the Smc (Gröschel et al., 2020).

We also profiled the potential pathogenic characteristics of Smc strains. Smc strains harbor a rich virulence-associated gene pool, including 17 virulence classes. The core virulence genes are related to the functions of adherence, colonization and invasion, biofilm formation, immune evasion and proteolysis. Both clinical and environmental Smc strains showed similar distributions of potential virulence-associated genes, which is consistent with the prior studies (Adamek et al., 2014; Mercier-Darty et al., 2020) and demonstrates the complexity of its pathogenic mechanisms. It is worth mentioning that a variety of Smc species exhibit the presence of T6SS-coding genes that are not anticipated in strains of *S. maltophilia*. The T6SS is a mechanism employed by bacterial cells to facilitate communication with their external environment and has resemblances to the piercing apparatus in bacteriophages. *P. aeruginosa* employs the T6SS for the purpose of delivering toxins to adjacent pathogens and transporting protein effectors to host cells (Chen et al., 2015). Similarly, several types of gut microbiota, such as *Salmonella Typhimurium* and *Vibrio cholerae*, can enhance colonization by utilizing the T6SS, thereby increasing their pathogenicity (Sana et al., 2016; Fast et al., 2018). This implies that species other than *S. maltophilia* could emerge as pathogens and cause disease in humans, and infection is likely to occur primarily due to a compromised anti-infective response in infected patients (Lira et al., 2017).

Swimming motility is associated with upregulation of virulence and induction of host defense mechanisms (Kazmierczak et al., 2015) and it also plays a significant role in causing skin infections (Zegadło et al., 2023). Our study indicated that *S. sepilia* exhibits stronger motility, which consistent with the predictions of virulence-associated genes, as *S. sepilia* contains a high number of genes encoding flagella and type IV pili, which are known to be involved in regulating bacterial swimming (Chaban et al., 2015; Nieto et al., 2019). Meanwhile, *S. maltophilia* has tenacious biofilm formation ability and can form a stable biofilm within 24 hours (Sun et al., 2016; Pompilio et al., 2020). Further biofilm formation ability assays in this study suggested that both *S. sepilia* and *S. maltophilia* have a greater ability to survive and colonize surfaces, potentially leading to a competitive advantage (Isom et al., 2022). A ANSELM prospective multicenter study reveals that there is a strong association between high-level biofilm production and invasive infections caused by *S. maltophilia* (Pompilio et al., 2020). Researches on *P. aeruginosa* and *Escherichia coli* have also revealed that strains that cause severe infections exhibit a greater propensity for biofilm formation as compared to those without a pathogenic role (Soto et al., 2007; Mulcahy et al., 2014; Ageorges et al., 2020). These observations imply that there exists a discernible correlation between the organism’s ability to develop biofilms and its level of pathogenicity (Pompilio et al., 2020). It is remarkable that *S. sepilia* has an unimaginable ability for swimming motility and biofilm formation. Its type strain was isolated from blood cultures of hospitalized patients (Gautam et al., 2021), indicating its underestimated pathogenicity. According to the prevalence analysis, *S. sepilia* has become the dominant strain in Smc, which deserves more attention.

There is a consensus that *S. maltophilia* exhibits resistance to a majority of antibiotics, leaving only a limited number of clinically effective drugs available to combat this organism (Gil-Gil et al., 2020; Brooke, 2021). Our study of clinical Smc strains found that these strains exhibited a high sensitivity rate to trimethoprim/sulfamethoxazole and minocycline. This finding is similar to the global overall susceptibility reported in the recent SENTRY study (Gales et al., 2019). However, all 14 *S. geniculata* strains were found to be resistant to Ceftazidime, and the resistance rate was significantly higher than other species in Smc, but we did not find that *S. geniculata* has a special genotype for the prediction of resistance genes. This may be an effect due to their complex resistance mechanism, the drug resistance phenotype and genotype of Smc are not always consistent (Wang et al., 2021), and the antibiotic efflux pump system may play an important role (Sánchez and Martínez, 2015).

As above stated, this study provides a comprehensive overview of the population diversity, evolution, and pathogenic characteristics of the understudied pathogen Smc. The 24 species-level clades of SMC vary in geographical distribution and pathogenic potential. Our research indicates that S. geniculata and S. sepilia display worrying phenotypic characteristics related to drug resistance and virulence, respectively, but currently we know very little about these clinical mainstream species of Smc. To better address infections caused by Smc, further studies are required to assess and compare species-specific characteristics related to epidemiology, disease spectrum, resistance, and virulence of species of Smc. Likewise, research is also needed to set out to identify unambiguous molecular targets that could reliably distinguish between these pathogens at the species level. Proper species delimitation is essential for uncovering species-specific phenotypic characteristics and enhancing our understanding of this complex pathogen.

## Data availability statement

The original contributions presented in the study are included in the article/**Supplementary Material**. Further inquiries can be directed to the corresponding authors.

## Author contributions

KL: Data curation, Formal analysis, Investigation, Visualization, Writing – original draft, Methodology, Validation. KY: Formal analysis, Methodology, Validation, Writing – review & editing. ZH: Formal analysis, Methodology, Software, Writing – review & editing. XL: Data curation, Methodology, Writing – review & editing. LM: Data curation, Methodology, Writing – review & editing. XR: Data curation, Investigation, Validation, Writing – review & editing. XB: Methodology, Validation, Writing – review & editing. HG: Investigation, Methodology, Validation, Writing – review & editing. ZS: Data curation, Writing – review & editing. XNL: Conceptualization, Investigation, Methodology, Project administration, Supervision, Writing – review & editing. DW: Conceptualization, Funding acquisition, Investigation, Methodology, Project administration, Resources, Supervision, Writing – review & editing.
