# Supplementary material for: Stenotrophomonas maltophilia complex: insights into evolutionary relationships, global distribution and pathogenicity
Source: Front Cell Infect Microbiol. 2024 Jan 10;13:1325379. doi: 10.3389/fcimb.2023.1325379 (PMC10806987; doi:10.3389/fcimb.2023.1325379)
Supplement: Supplementary file 1 [file Table_1.pdf]

Stenotrophomonas maltophilia Complex: Insights into Evolutionary Relationships, Global Distribution and Pathogenicity

Kun Li[1,2], Keyi Yu[2], Zhenzhou Huang[3], Xiao Liu[2], Li Mei[4], Xiaodong Ren[1,2], Xuemei Bai[2], He Gao[2], Zhiwen Sun[2], Xiaoning Liu[1,], Duochun Wang[2,*]*

[1]School of Public Health, Lanzhou University, Lanzhou, China

[2]National Institute for Communicable Disease Control and Prevention, Chinese Center for Disease Control and Prevention, State Key Laboratory of Infectious Disease Prevention and Control, Beijing, China

[3]Hangzhou Center for Disease Control and Prevention, Hangzhou, China

[4]National Pathogen Resource Center, Chinese Center for Disease Control and Prevention, Beijing, China

*Correspondence:

Xiaoning Liu, liuxn@lzu.edu.cn

Duochun Wang, wangduochun@icdc.cn.

TABLE S1 List of 90 newly sequenced stains of *S. maltophilia*, their taxonomic classification by ANI, genome features along with isolation source

Strain	Classification by ANI	CDS	tRNA	GC%	Contigs	Genome size (bp)	Isolate date	Country	Sample source
SMYL1	*S. geniculata*	3505	54	66.63	32	3951482	2020	China	bile
SMYL10	*S. sepilia*	3584	72	66.68	58	4050075	2022	China	oropharyngeal swab
SMYL100	*P. hibiscicola*	3872	76	66.53	28	4359971	2023	China	sputum
SMYL101	*S. maltophilia*	3942	75	66.72	42	4446972	2023	China	sputum
SMYL102	*S. sepilia*	4037	74	66.42	34	4570124	2023	China	sputum
SMYL103	*S. maltophilia*	4321	73	66.38	57	4752237	2023	China	sputum
SMYL104	*S. maltophilia*	4550	73	66.33	42	5030460	2023	China	trachea tube
SMYL105	*P. hibiscicola*	3942	74	66.49	33	4436945	2023	China	sputum
SMYL107	*S. pavanii*	4050	71	67.45	39	4542776	2023	China	sputum
SMYL109	*S. maltophilia*	4313	74	66.38	53	4798022	2023	China	trachea tube
SMYL11	Genospecies 1	4231	72	66.38	31	4710808	2022	China	sputum
SMYL111	*S. maltophilia*	4329	76	66.39	57	4820555	2023	China	trachea tube
SMYL112	*P. hibiscicola*	3923	77	66.44	30	4380634	2023	China	sputum
SMYL113	*S. maltophilia*	4233	73	66.37	63	4747584	2023	China	sputum
SMYL12	*S. sepilia*	3935	73	66.77	39	4411314	2022	China	sputum
SMYL13	*S. muris*	4041	73	67.00	42	4486376	2022	China	sputum
SMYL14	*S. maltophilia*	4295	76	66.44	76	4781637	2022	China	trachea tube
SMYL15	*S. sepilia*	3905	76	66.74	29	4381745	2022	China	oropharyngeal swab
SMYL16	*S. maltophilia*	4316	74	66.39	64	4790790	2022	China	sputum
SMYL19	*S. geniculata*	4043	73	66.60	41	4509011	2022	China	sputum
SMYL2	*S. maltophilia*	4451	74	66.14	43	4920167	2019	China	sputum
SMYL20	Genospecies 1	4161	71	66.42	55	4636787	2022	China	sputum
SMYL21	*S. maltophilia*	4241	80	66.62	67	4689707	2022	China	sputum

SMYL22	*S. geniculata*	4108	71	66.48	35	4583091	2022	China	sputum
SMYL23	*S. muris*	4038	75	67.00	37	4483387	2022	China	sputum
SMYL25	*S. maltophilia*	4220	75	66.47	74	4676726	2022	China	sputum
SMYL26	*S. maltophilia*	4300	75	66.44	76	4790720	2022	China	trachea tube
SMYL27	*S. sepilia*	4033	73	66.56	41	4477620	2022	China	oropharyngeal swab
SMYL28	Genospecies 7	4343	73	66.26	59	4785883	2022	China	sputum
SMYL29	*S. maltophilia*	4291	71	66.44	66	4776737	2022	China	trachea tube
SMYL3	*S. maltophilia*	4331	74	66.51	55	4781988	2021	China	feces
SMYL33	*S. maltophilia*	4472	76	66.47	68	4921012	2021	China	feces
SMYL34	*S. maltophilia*	4301	76	66.44	78	4792467	2022	China	trachea tube
SMYL36	Genospecies 2	4235	73	66.41	39	4686047	2022	China	sputum
SMYL37	*S. muris*	4144	72	66.97	47	4552326	2022	China	sputum
SMYL38	*S. muris*	4086	71	66.95	43	4531590	2022	China	sputum
SMYL4	*S. geniculata*	3732	73	66.59	40	4180322	2022	China	sputum
SMYL40	*S. maltophilia*	4113	73	66.73	73	4566801	2022	China	sputum
SMYL41	Genospecies 4	4413	76	66.54	34	4928835	2022	China	sputum
SMYL42	*P. hibiscicola*	3925	73	66.56	40	4378224	2022	China	sputum
SMYL44	*S. sepilia*	3918	65	66.55	92	4336966	2022	China	sputum
SMYL49	*S. geniculata*	4049	72	66.57	32	4526138	2022	China	sputum
SMYL5	*S. maltophilia*	4405	68	66.49	59	4844343	2021	China	feces
SMYL51	*S. maltophilia*	4027	73	66.68	55	4500621	2022	China	sputum
SMYL52	*S. maltophilia*	4068	76	66.54	76	4560644	2022	China	sputum
SMYL54	*S. pavanii*	3716	73	67.50	58	4179212	2022	China	sputum
SMYL55	*S. maltophilia*	4272	81	66.39	56	4759495	2022	China	sputum
SMYL56	*S. sepilia*	4119	73	66.62	61	4579512	2022	China	sputum
SMYL57	*S. sepilia*	4005	74	66.76	53	4517388	2022	China	sputum

SMYL58	*S. maltophilia*	4228	75	66.54	80	4662512	2022	China	sputum
SMYL59	*S. maltophilia*	4148	75	66.56	62	4646047	2022	China	sputum
SMYL6	*S. maltophilia*	4552	77	66.47	54	5002246	2021	China	feces
SMYL60	*S. muris*	3936	72	67.08	43	4381837	2022	China	sputum
SMYL61	*S. geniculata*	4152	73	66.58	41	4632844	2022	China	sputum
SMYL62	*S. geniculata*	4117	70	66.46	45	4613043	2022	China	drainage fluid
SMYL63	*S. sepilia*	3705	75	66.77	37	4155600	2022	China	drainage fluid
SMYL64	*S. geniculata*	4051	66	66.14	28	4465299	2022	China	sputum
SMYL67	*S. sepilia*	3992	73	66.65	37	4477230	2022	China	wound
SMYL68	*S. geniculata*	3982	72	66.64	44	4464001	2022	China	sputum
SMYL69	*S. maltophilia*	4283	74	66.61	63	4694432	2022	China	sputum
SMYL7	Genospecies 1	4481	72	66.28	63	4945492	2021	China	feces
SMYL70	*S. maltophilia*	4295	74	66.43	72	4779658	2022	China	trachea tube
SMYL71	*S. sepilia*	3989	73	66.56	54	4463887	2022	China	sputum
SMYL73	*S. geniculata*	3880	65	66.60	38	4319827	2022	China	sputum
SMYL74	*S. maltophilia*	4030	81	66.69	66	4496452	2022	China	sputum
SMYL75	*S. maltophilia*	3986	77	66.71	64	4453286	2022	China	sputum
SMYL76	*S. geniculata*	3771	71	66.56	30	4214529	2022	China	sputum
SMYL78	*S. sepilia*	3768	69	66.67	37	4173853	2023	China	urine
SMYL79	*S. maltophilia*	4300	76	66.44	80	4785147	2023	China	unknown
SMYL8	Genospecies 1	4361	77	66.23	63	4840340	2022	China	sputum
SMYL80	*S. maltophilia*	4124	76	66.60	72	4595442	2023	China	drainage fluid
SMYL82	Genospecies 5	4120	73	66.65	44	4639131	2023	China	blood
SMYL83	*S. pavanii*	4104	73	67.09	65	4605017	2023	China	urine
SMYL84	*S. maltophilia*	4318	74	66.59	86	4730691	2023	China	sputum
SMYL85	*S. maltophilia*	4297	72	66.44	80	4786985	2023	China	sputum

SMYL86	Genospecies 1	4269	69	66.36	42	4667427	2023	China	sputum
SMYL87	*S. maltophilia*	4176	75	66.54	88	4639505	2023	China	sputum
SMYL88	*S. maltophilia*	4306	75	66.44	74	4796557	2023	China	trachea tube
SMYL89	Genospecies 3	3978	77	66.62	43	4468730	2023	China	sputum
SMYL9	*S. geniculata*	3730	72	66.63	36	4204063	2022	China	sputum
SMYL90	*S. maltophilia*	3969	75	66.69	70	4488752	2023	China	wound
SMYL91	*S. maltophilia*	4338	79	66.38	58	4790115	2023	China	sputum
SMYL92	*S. maltophilia*	4211	75	66.45	60	4723800	2023	China	sputum
SMYL93	*S. sepilia*	4035	76	66.58	46	4536450	2023	China	sputum
SMYL94	*S. maltophilia*	4096	79	66.50	72	4594656	2023	China	sputum
SMYL95	*S. maltophilia*	4261	73	66.42	56	4674604	2023	China	sputum
SMYL96	*S. pavanii*	4157	75	67.13	74	4655521	2023	China	sputum
SMYL97	*S. geniculata*	3802	70	66.58	12	4247608	2023	China	sputum
SMYL98	*S. geniculata*	3971	71	66.58	39	4443435	2023	China	sputum
SMYL99	*S. africana*	4389	73	66.04	21	4850490	2023	China	sputum

TABLE S2 List of 668 *S. maltophilia* genomes from NCBI, their assembly accession, taxonomic classification by ANI, genome features along with isolation source

Assembly accession	Strain	Classification by ANI	CDS	tRNA	GC%	Contigs	Size (bp)	BioSample	Isolate date	Country	Sample source
GCA_000020665.1	R551-3	Genospecies 10	4043	81	66.30	1	4573969	SAMN00623065	unknown	USA	poplar Populustrichocarpa
GCA_000072485.1	K279a	*S. maltophilia*	4412	79	66.32	1	4851126	SAMEA1705934	1998	United Kingdom	blood
GCA_000223885.1	JV3	Genospecies 11	4071	78	66.89	1	4544477	SAMN02261377	unknown	unknown	plants root
GCA_000237025.2	RR-10	*S. geniculata*	4192	110	66.34	158	4660562	SAMN02471024	2010	China	rice root
GCA_000284595.1	D457	*S. muris*	4312	79	66.75	1	4769156	SAMEA2272378	unknown	Spain	unknown
GCA_000287935.1	Ab55555	*S. maltophilia*	4456	75	66.12	6	4918930	SAMN02596924	unknown	unknown	unknown
GCA_000308335.1	PML168	Genospecies 8	3934	66	66.57	97	4403876	SAMEA2272452	2013	United Kingdom	rock pool
GCA_000344215.1	EPM1	*S. maltophilia*	4347	71	66.37	1	4787769	SAMN02471395	unknown	Portugal	unknown
GCA_000346445.1	AU12-09	*S. sepilia*	4110	70	66.40	125	4547300	SAMN02469852	2012	Australia	catheter tip
GCA_000499565.1	stmalt0377	*S. maltophilia*	4224	67	66.43	120	4620840	SAMEA3138820	unknown	unknown	unknown
GCA_000543365.1	5BA-I-2	*S. indicatrix*	4064	71	66.40	4	4568054	SAMN02641498	2007	Czech Republic	soil
GCA_000611735.2	M30	*S. muris*	4477	71	66.37	193	4902008	SAMN02592618	2009	Spain	decubitus ulcer
GCA_000742995.1	13637	*S. maltophilia*	4566	74	66.15	1	4989312	SAMN02874005	1961	USA	Oropharyngeal
GCA_000758465.1	53	Genospecies 1	4148	69	66.34	127	4637887	SAMN03067892	2010	United Kingdom	hospital isolate
GCA_000834105.1	ZBG7B	*S. pennii*	3598	59	66.33	145	4065399	SAMN03280975	2014	France	soil
GCA_000973145.1	JMNMN1	*S. maltophilia*	6675	41	66.42	30	4150255	SAMN03393603	2013	India	urine
GCA_000978875.1	UV74	*S. muris*	4471	74	66.65	179	4889583	SAMN03076212	2009	Spain	vascular ulcer
GCA_001068765.1	1162_SMAL	*S. sepilia*	3919	60	66.73	108	4392826	SAMN03197119	unknown	USA	unknown

GCA_001068915.1	1253_SMAL	*S. maltophilia*	4392	62	66.23	102	4833739	SAMN03197220	unknown	USA	unknown
GCA_001069615.1	1305.rep2_SMAL	*S. maltophilia*	4629	57	66.21	158	5024838	SAMN03197279	unknown	USA	unknown
GCA_001069645.1	131_SMAL	Genospecies 1	4346	71	66.02	71	4794818	SAMN03197286	unknown	USA	unknown
GCA_001070865.1	32_SMAL	Genospecies 1	4791	66	65.70	104	5198613	SAMN03197510	unknown	USA	unknown
GCA_001070945.1	36_SMAL	Genospecies 1	4778	54	65.73	178	5186304	SAMN03197550	unknown	USA	unknown
GCA_001071045.1	411_SMAL	*S. geniculata*	4303	72	66.23	33	4779756	SAMN03197602	unknown	USA	unknown
GCA_001071065.1	419_SMAL	*S. geniculata*	4311	62	66.25	82	4770917	SAMN03197610	unknown	USA	unknown
GCA_001071115.1	447_SMAL	*S. muris*	4372	73	66.54	47	4802736	SAMN03197640	unknown	USA	unknown
GCA_001071195.1	498_SMAL	*S. maltophilia*	4485	67	66.31	114	4868454	SAMN03197690	unknown	USA	unknown
GCA_001071285.1	539_SMAL	*S. maltophilia*	4469	70	66.02	114	4953618	SAMN03197735	unknown	USA	unknown
GCA_001071335.1	543_SMAL	Genospecies 1	4790	70	65.68	126	5203047	SAMN03197741	unknown	USA	unknown
GCA_001071345.1	544_SMAL	Genospecies 1	4780	69	65.72	112	5188184	SAMN03197742	unknown	USA	unknown
GCA_001071655.1	179_SMAL	*S. geniculata*	4043	58	66.45	67	4533188	SAMN03197369	unknown	USA	unknown
GCA_001071815.1	22_SMAL	*S. geniculata*	3982	76	66.51	38	4457527	SAMN03197412	unknown	USA	unknown
GCA_001072195.1	305_SMAL	*S. maltophilia*	4329	70	66.38	33	4732922	SAMN03197497	unknown	USA	unknown
GCA_001072535.1	363_SMAL	*S. maltophilia*	4325	46	66.38	136	4721538	SAMN03197553	unknown	USA	unknown
GCA_001072635.1	372_SMAL	*S. maltophilia*	4571	62	66.19	162	4995632	SAMN03197563	unknown	USA	unknown
GCA_001072845.1	416_SMAL	Genospecies 1	4817	68	65.69	99	5214364	SAMN03197607	unknown	USA	unknown
GCA_001072915.1	424_SMAL	Genospecies 1	4795	74	65.70	68	5202479	SAMN03197617	unknown	USA	unknown
GCA_001073045.1	453_SMAL	*S. geniculata*	4303	72	66.23	28	4779898	SAMN03197645	unknown	USA	unknown
GCA_001073225.1	517_SMAL	*S. geniculata*	4082	54	66.42	108	4569872	SAMN03197711	unknown	USA	unknown
GCA_001274595.1	Patient 2, ISMMS3	Genospecies 13	4285	81	66.65	1	4804002	SAMN03389650	2013	USA	blood
GCA_001274655.1	ISMMS2	*S. geniculata*	4015	78	66.45	1	4509724	SAMN03389647	2013	USA	blood
GCA_001274675.1	ISMMS2R	*S. geniculata*	4015	78	66.45	1	4509724	SAMN03389649	2013	USA	blood

GCA_001275075.1	ISMMS5	*S. muris*	4319	80	66.64	18	4784825	SAMN03389652	2013	USA	blood
GCA_001275085.1	ISMMS4	*S. maltophilia*	4327	79	66.20	3	4752108	SAMN03389651	2013	USA	blood
GCA_001275095.1	ISMMS6	*S. pavanii*	4502	78	66.92	10	4832466	SAMN03389653	2013	USA	blood
GCA_001276355.1	B4	Genospecies 6	3932	39	66.65	180	4334733	SAMN03753636	2005	Australia	lungs of CF
GCA_001286925.1	Mecca	*S. sepilia*	3966	75	66.51	34	4386843	SAMEA3751020	2014	Saudi Arabia	unknown
GCA_001431675.1	LMG 22072T	*S. africana*	4076	70	66.32	173	4512166	SAMN04207869	1994	Democratic Republic of the Congo	cerebrospinal fluid
GCA_001499695.1	BurE1	*S. maltophilia*	4029	75	66.57	48	4504590	SAMEA3309461	2008	Burkina Faso	soil
GCA_001499715.1	BurA1	*P. hibiscicola*	3954	67	66.57	64	4360660	SAMEA3309460	2008	Burkina Faso	soil
GCA_001542395.1	PG157	*S. maltophilia*	4529	79	66.50	76	4949420	SAMN04260440	2011	Slovenia	perineum
GCA_001591205.1	NBRC 14161	*S. maltophilia*	4530	69	66.19	131	4936723	SAMD00046746	unknown	unknown	unknown
GCA_001619675.1	F2	*S. muris*	4241	74	66.65	172	4752918	SAMN04386403	2011	Malaysia	Ulu Slim Hot Spring
GCA_001651505.1	ISMMS7	*S. pavanii*	4226	82	66.86	2	4702773	SAMN03389654	2013	USA	blood
GCA_001676295.1	Sm32COP	*S. sepilia*	4036	74	66.40	45	4548960	SAMN05188742	2010	France	horse manure
GCA_001676315.1	Sm41DVV	Genospecies 6	3679	71	66.89	26	4139723	SAMN05188789	2010	France	horse manure
GCA_001676375.1	SmF3	*S. maltophilia*	4113	78	66.52	77	4595297	SAMN05189120	2007	France	cattle manure
GCA_001676395.1	SmF22	*S. sepilia*	4158	72	66.37	64	4583062	SAMN05190064	2007	France	cattle manure
GCA_001676435.1	SmCVFa1	Genospecies 6	3786	74	66.81	30	4264176	SAMN05190067	2012	France	cattle manure

GCA_001676445.1	SmSOFb1	*S. sepilia*	4086	82	66.46	93	4483386	SAMN05190066	2012	France	horse manure
GCA_001693455.1	SBo1	Genospecies 10	4337	80	66.14	23	4800701	SAMN05294119	2015	Greece	gut
GCA_001858265.1	R5G	Genospecies 1	4527	72	65.89	79	4992669	SAMN05413110	2013	Malaysia	Dentine Caries
GCA_001866065.1	DT1	*S. geniculata*	4027	73	66.51	64	4526952	SAMN05915699	2013	China	Tetracycline contaminated soil
GCA_001997185.1	ATCC 13637	*S. maltophilia*	4527	73	66.18	182	4951541	SAMN05721779	unknown	unknown	unknown
GCA_002025605.1	AA1	Genospecies 17	4123	84	67.38	1	4663337	SAMN06130959	2012	USA	root
GCA_002138415.1	OUC_Est10	Genospecies 21	4182	78	66.25	1	4668743	SAMN04992827	2013	China	soil
GCA_002189545.2	AB550	*S. muris*	4541	81	66.51	1	4943426	SAMN06678536	2016	Australia	water
GCA_002189565.1	SB275	*S. maltophilia*	4125	75	66.61	46	4573126	SAMN06678627	2016	Australia	water
GCA_002205155.1	M16356	*S. maltophilia*	4369	61	66.19	169	4825809	SAMN07237140	2006	USA	blood
GCA_002205165.1	13146	Genospecies 15	3894	58	66.63	60	4444179	SAMN07237143	2007	USA	CF sputum
GCA_002205175.1	163919	*S. maltophilia*	4310	66	66.33	148	4695069	SAMN07237142	2014	USA	blood
GCA_002205215.1	594	*S. sepilia*	4018	66	66.63	119	4484627	SAMN07237146	2014	USA	rectal swab
GCA_002205225.1	S2722A9	*S. muris*	4452	64	66.61	176	4867831	SAMN07237139	2015	USA	rectal swab
GCA_002205245.1	910	*S. africana*	4241	66	66.15	132	4712083	SAMN07237145	2015	USA	blood
GCA_002205275.1	1038	*S. muris*	4239	62	66.74	146	4695098	SAMN07237144	2015	USA	rectal swab
GCA_002205295.1	7141307	*S. maltophilia*	4271	63	66.39	129	4725889	SAMN07237141	2011	USA	thracheal aspirate
GCA_002208885.2	FDAARGOS_325	*S. maltophilia*	4448	81	66.25	1	4851512	SAMN06173338	2015	USA	eye
GCA_002277735.1	DF07	*S. sepilia*	4345	77	66.23	154	4801842	SAMN07253293	2017	USA	soil
GCA_002738625.1	ZC_2005	*S. maltophilia*	4443	73	66.26	96	4890599	SAMN06019650	2005	Italy	lung
GCA_002738635.1	ZC_2007	*S. maltophilia*	3932	73	66.63	79	4355949	SAMN06019652	2007	Italy	lung

GCA_002738665.1	ZC_2006	*S. maltophilia*	3934	73	66.63	109	4355118	SAMN06019651	2006	Italy	lung
GCA_002738675.1	ZC_2004	*S. maltophilia*	4101	76	66.58	123	4559876	SAMN06019649	2004	Italy	lung
GCA_002738705.1	ZC_2008	*S. maltophilia*	3932	73	66.63	67	4354099	SAMN06019653	2008	Italy	lung
GCA_002738715.1	ZC_2009	*S. maltophilia*	3932	73	66.63	104	4355770	SAMN06019654	2009	Italy	lung
GCA_002738725.1	ZC_2010	*S. maltophilia*	4418	75	66.28	107	4871504	SAMN06019655	2010	Italy	lung
GCA_002738765.1	ZC_2011	*S. maltophilia*	4480	71	66.24	181	4905486	SAMN06019656	2011	Italy	lung
GCA_002738785.1	ZC_2012_STM2	*S. maltophilia*	3934	72	66.63	94	4354314	SAMN06019658	2012	Italy	lung
GCA_002738805.1	ZC_2013_STM2	*S. maltophilia*	3924	73	66.63	86	4348950	SAMN06019660	2013	Italy	lung
GCA_002738815.1	ZC_2013_STM1	*S. maltophilia*	4435	74	66.29	105	4888565	SAMN06019659	2013	Italy	lung
GCA_002738825.1	ZC_2012_STM1	*S. maltophilia*	4478	75	66.25	109	4909153	SAMN06019657	2012	Italy	lung
GCA_002738845.1	ZC_2014	*S. maltophilia*	3921	73	66.64	84	4346966	SAMN06019661	2014	Italy	lung
GCA_002738885.1	CV_2003_STM2	*S. maltophilia*	4471	77	66.32	91	4869364	SAMN06019663	2003	Italy	lung
GCA_002738905.1	CV_2003_STM1	*S. africana*	4394	73	66.18	132	4833118	SAMN06019662	2003	Italy	lung
GCA_002738915.1	CV_2005	*S. maltophilia*	4464	76	66.31	114	4868668	SAMN06019664	2005	Italy	lung
GCA_002738925.1	CV_2006_STM1	*S. maltophilia*	4401	76	66.35	130	4804712	SAMN06019665	2006	Italy	lung
GCA_002738965.1	CV_2006_STM2	*S. maltophilia*	4394	78	66.35	111	4800283	SAMN06019666	2006	Italy	lung
GCA_002738985.1	CV_2008	*S. maltophilia*	4392	73	66.35	106	4801064	SAMN06019668	2008	Italy	lung
GCA_002739005.1	TG_2006_S	*S. sepilia*	3877	73	66.79	63	4348898	SAMN06019683	2006	Italy	lung

	TM1										
GCA_002739015.1	CV_2007	*S. maltophilia*	4395	73	66.35	137	4797657	SAMN06019667	2007	Italy	lung
GCA_002739025.1	TG_2005	*S. sepilia*	3876	73	66.79	81	4350163	SAMN06019682	2005	Italy	lung
GCA_002739065.1	TG_2006_STM2	*S. sepilia*	3875	72	66.79	70	4348675	SAMN06019684	2006	Italy	lung
GCA_002739075.1	TG_2007_STM1	*S. sepilia*	3864	72	66.80	85	4342024	SAMN06019685	2007	Italy	lung
GCA_002739095.1	TG_2007_STM2	*S. sepilia*	3876	72	66.79	70	4350360	SAMN06019686	2007	Italy	lung
GCA_002739105.1	TG_2009_STM1	*S. sepilia*	3878	72	66.79	98	4344434	SAMN06019689	2009	Italy	lung
GCA_002739145.1	TG_2012_STM2	*S. sepilia*	3877	70	66.79	85	4345979	SAMN06019692	2012	Italy	lung
GCA_002739155.1	Fma_2005_STM1	*S. maltophilia*	4171	73	66.49	103	4615168	SAMN06019696	2005	Italy	lung
GCA_002739175.1	Fma_2008_STM2	*S. maltophilia*	4322	73	66.45	80	4774285	SAMN06019699	2008	Italy	lung
GCA_002739195.1	Fma_2009	*S. maltophilia*	4205	75	66.44	98	4656954	SAMN06019700	2009	Italy	lung
GCA_002739225.1	Fma_2010_STM1	*S. maltophilia*	4209	74	66.46	109	4650032	SAMN06019701	2010	Italy	lung
GCA_002739235.1	Fma_2010_STM2	*S. maltophilia*	4206	73	66.46	105	4651210	SAMN06019702	2010	Italy	lung
GCA_002739255.1	AV_2010	*S. maltophilia*	4153	77	66.42	135	4616950	SAMN06019708	2010	Italy	lung
GCA_002739275.1	AV_2011	*S. maltophilia*	4437	74	66.27	146	4869806	SAMN06019709	2011	Italy	lung
GCA_002739305.1	AV_2012_STM1	*S. maltophilia*	4490	75	66.27	136	4906760	SAMN06019710	2012	Italy	lung

GCA_002739315.1	GC_2008_colA	*S. maltophilia*	4304	75	66.49	95	4735169	SAMN06019713	2008	Italy	lung
GCA_002739345.1	GC_2008_colB	*S. maltophilia*	4301	77	66.49	95	4736315	SAMN06019714	2008	Italy	lung
GCA_002739375.1	GC_2012_STM2	*S. maltophilia*	4300	76	66.05	92	4740614	SAMN06019719	2012	Italy	lung
GCA_002739395.1	GC_2011	*S. maltophilia*	4303	77	66.49	110	4735249	SAMN06019717	2011	Italy	lung
GCA_002739405.1	GC_2013_STM1	*S. maltophilia*	4202	75	66.49	105	4618695	SAMN06019720	2013	Italy	lung
GCA_002739445.1	GC_2013_STM2	*S. maltophilia*	4297	74	66.49	96	4738508	SAMN06019721	2013	Italy	lung
GCA_002739455.1	MC_2010_STM1	Genospecies 2	4432	70	66.28	91	4882242	SAMN06019725	2010	Italy	lung
GCA_002739465.1	MC_2010_STM2	*S. sepilia*	3918	70	66.81	88	4378589	SAMN06019726	2010	Italy	lung
GCA_002739505.1	MC_2011_STM2	*S. sepilia*	3923	74	66.81	97	4377825	SAMN06019728	2011	Italy	lung
GCA_002739525.1	BB_2011	*S. maltophilia*	4383	76	66.41	106	4823438	SAMN06019733	2011	Italy	lung
GCA_002739535.1	CV_2010	*S. maltophilia*	4391	75	66.36	136	4797547	SAMN06019669	2010	Italy	lung
GCA_002739555.1	CV_2013	*S. muris*	4300	72	66.42	148	4790996	SAMN06019670	2013	Italy	lung
GCA_002739595.1	MS_2003_STM1	*S. maltophilia*	4359	73	66.15	141	4842401	SAMN06019671	2003	Italy	lung
GCA_002739605.1	MS_2006	*S. maltophilia*	4500	71	66.21	175	4936554	SAMN06019673	2006	Italy	lung
GCA_002739625.1	MS_2008	*S. geniculata*	4026	71	66.51	68	4463128	SAMN06019674	2008	Italy	lung
GCA_002739665.1	MS_2010	*S. maltophilia*	4494	77	66.32	130	4937359	SAMN06019676	2010	Italy	lung
GCA_002739675.1	MS_2009	Genospecies 2	4295	72	66.32	123	4796729	SAMN06019675	2009	Italy	lung

GCA_002739685.1	MS_2011	*S. maltophilia*	4509	77	66.32	123	4939324	SAMN06019677	2011	Italy	lung
GCA_002739715.1	MS_2012	*S. maltophilia*	4468	77	66.33	100	4905248	SAMN06019678	2012	Italy	lung
GCA_002739745.1	MS_2013	*S. maltophilia*	4444	76	66.15	96	4939558	SAMN06019679	2013	Italy	lung
GCA_002739755.1	MS_2014	*S. maltophilia*	4527	73	66.25	142	4975192	SAMN06019680	2014	Italy	lung
GCA_002739775.1	TG_2004	*S. maltophilia*	4059	78	66.63	96	4530396	SAMN06019681	2004	Italy	lung
GCA_002739785.1	TG_2008_STM1	*S. sepilia*	3877	72	66.79	82	4340861	SAMN06019687	2008	Italy	lung
GCA_002739825.1	TG_2008_STM2	*S. sepilia*	3873	73	66.79	78	4346012	SAMN06019688	2008	Italy	lung
GCA_002739835.1	TG_2009_STM2	*S. sepilia*	3871	70	66.80	75	4347965	SAMN06019690	2009	Italy	lung
GCA_002739855.1	TG_2012_STM1	*S. sepilia*	3878	70	66.79	72	4349116	SAMN06019691	2012	Italy	lung
GCA_002739875.1	TG_2013	*S. sepilia*	3882	72	66.79	105	4345785	SAMN06019693	2013	Italy	lung
GCA_002739905.1	TG_2014_STM1	*S. sepilia*	3884	72	66.80	98	4344642	SAMN06019694	2014	Italy	lung
GCA_002739915.1	Fma_2007	*S. maltophilia*	4110	77	66.43	134	4604066	SAMN06019698	2007	Italy	lung
GCA_002739945.1	TG_2014_STM2	*S. sepilia*	3910	73	66.79	109	4368250	SAMN06019695	2014	Italy	lung
GCA_002739955.1	Fma_2005_STM2	*S. maltophilia*	4160	73	66.49	88	4612173	SAMN06019697	2005	Italy	lung
GCA_002739985.1	Fma_2011	*S. maltophilia*	4203	73	66.46	119	4648324	SAMN06019703	2011	Italy	lung
GCA_002739995.1	Fma_2012	*S. maltophilia*	4208	72	66.46	117	4650171	SAMN06019704	2012	Italy	lung
GCA_002740025.1	AV_2009	Genospecies 2	4351	70	66.34	89	4827143	SAMN06019707	2009	Italy	lung
GCA_002740035.1	AV_2006	*S. maltophilia*	4188	77	66.40	123	4651562	SAMN06019706	2006	Italy	lung
GCA_002740055.1	AV_2013	*S. maltophilia*	4491	75	66.27	98	4913731	SAMN06019712	2013	Italy	lung

GCA_002740085.1	AV_2012_S TM2	*S. maltophilia*	4551	77	66.30	99	5015330	SAMN06019711	2012	Italy	lung
GCA_002740095.1	GC_2010	*S. maltophilia*	4302	76	66.49	97	4739009	SAMN06019716	2010	Italy	lung
GCA_002740105.1	GC_2014_S TM1	*S. maltophilia*	4210	77	66.48	92	4629761	SAMN06019722	2014	Italy	lung
GCA_002740115.1	GC_2012_S TM1	*S. maltophilia*	4290	76	66.50	104	4731946	SAMN06019718	2012	Italy	lung
GCA_002740165.1	MC_2009	*S. maltophilia*	4454	74	66.28	89	4887793	SAMN06019724	2009	Italy	lung
GCA_002740175.1	GC_2014_S TM2	*S. maltophilia*	4225	77	66.52	90	4686641	SAMN06019723	2014	Italy	lung
GCA_002740185.1	MC_2011_S TM1	*S. maltophilia*	4405	75	66.44	152	4794949	SAMN06019727	2011	Italy	lung
GCA_002740215.1	MC_2012	*S. sepilia*	3922	74	66.81	89	4380555	SAMN06019729	2012	Italy	lung
GCA_002740245.1	MC_2014	*S. sepilia*	3929	73	66.81	81	4379493	SAMN06019731	2014	Italy	lung
GCA_002740255.1	MC_2013	*S. sepilia*	3926	74	66.81	94	4381248	SAMN06019730	2013	Italy	lung
GCA_002740265.1	BB_2010	*S. maltophilia*	4118	76	66.55	116	4606510	SAMN06019732	2010	Italy	lung
GCA_002740285.1	BB_2012	*S. maltophilia*	4395	78	66.40	106	4825841	SAMN06019734	2012	Italy	lung
GCA_002740325.1	BB_2013	*S. maltophilia*	4276	77	66.56	92	4763796	SAMN06019735	2013	Italy	lung
GCA_002740345.1	SanG_2010	*S. maltophilia*	4383	79	66.43	115	4789465	SAMN06019736	2010	Italy	lung
GCA_002740355.1	SanG_2011	*S. maltophilia*	4374	76	66.63	97	4758010	SAMN06019737	2011	Italy	lung
GCA_002740365.1	SanG_2012	*S. maltophilia*	4507	73	66.12	103	4909273	SAMN06019738	2012	Italy	lung
GCA_002740395.1	SanG_2013	*S. maltophilia*	4378	75	66.26	80	4801791	SAMN06019739	2013	Italy	lung
GCA_002740425.1	AV_2005	*S. maltophilia*	4245	77	66.34	132	4720290	SAMN06019705	2005	Italy	lung
GCA_002798885.1	NS26	Genospecies 10	4170	74	66.21	15	4683398	SAMN06645022	1999	Germany	Environment
GCA_002798925.1	EA63	Genospecies 3	4407	73	66.00	7	4879275	SAMN06645021	2000	Germany	Sewage
GCA_002798945.1	EA21	*S. africana*	4262	72	66.20	6	4726489	SAMN06645020	2000	Germany	Sewage

GCA_002798965.1	EP5	*S. indicatrix*	4104	70	66.35	4	4594415	SAMN06645018	1995	Germany	Rhizosphere of oilseed rape
GCA_002798995.1	EP20	*S. lactitubi*	4099	70	66.04	12	4618818	SAMN06645002	1998	Germany	Rhizosphere of potato
GCA_002799015.1	EA23	*S. maltophilia*	4284	73	66.43	26	4746537	SAMN06644988	1999	Germany	Eye-care solution
GCA_002799025.1	PS5	*S. indicatrix*	4105	70	66.35	4	4594709	SAMN06644956	1996	Germany	Rhizosphere of oilseed rape
GCA_002799055.1	EP13	*S. maltophilia*	4287	73	66.43	24	4749990	SAMN06644924	1995	Germany	Rhizosphere of rape
GCA_002799065.1	E824	Genospecies 10	4519	71	65.86	11	5036145	SAMN06644907	1993	Spain	blood
GCA_002799095.1	C357	Genospecies 1	4336	73	66.18	14	4804814	SAMN06644905	1991	Spain	urine
GCA_002799105.1	E539	*S. maltophilia*	4069	76	66.55	14	4541906	SAMN06644906	1993	Spain	Pus from a wound
GCA_002799135.1	E861	*S. maltophilia*	4185	73	66.39	28	4652436	SAMN06644904	1994	Spain	sputum
GCA_002799145.1	D388	*S. maltophilia*	4186	73	66.39	27	4654219	SAMN06644903	1991	Spain	urine
GCA_002799155.1	E301	Genospecies 4	3984	71	66.81	2	4422561	SAMN06644901	1992	Spain	urine
GCA_002799165.1	G51	Genospecies 3	4397	75	66.11	5	4846973	SAMN06644900	1994	Spain	blood
GCA_002799215.1	E999	*S. sepilia*	3908	70	66.75	8	4408302	SAMN06644898	1993	Spain	Respiratory secretion
GCA_002799225.1	E759	*S. geniculata*	4096	69	66.46	3	4540892	SAMN06644893	1993	Spain	sputum
GCA_002799235.1	E729	*S. muris*	4602	71	66.56	9	4999783	SAMN06644891	1993	Spain	urine
GCA_002799245.1	EA1	Genospecies 5	4244	71	66.55	11	4746430	SAMN06644928	1996	Germany	Brackish water
GCA_002799295.1	EA22	Genospecies 3	4268	73	66.16	7	4753827	SAMN06644927	1999	Germany	Sewage
GCA_002847385.1	CSM2	Genospecies 11	4235	79	66.59	1	4739049	SAMN08161503	2016	Mexico	Laboratory sink
GCA_002951115.1	FDAARGO	*S. maltophilia*	4416	80	66.26	1	4820221	SAMN03996266	2013	USA	respiratory Cx

	S_92										
GCA_003006435.1	SJTH1	Genospecies 7	4459	77	65.94	1	4932325	SAMN08643285	2017	China	wastewater
GCA_003015035.1	XH.SM.1	Genospecies 3	4076	72	66.62	145	4561842	SAMN08093584	2004	China	liver
GCA_003030985.1	W18	*S. africana*	4286	80	66.13	1	4738432	SAMN08798397	2012	China	oil contaminated soil
GCA_003057835.2	17307	*S. maltophilia*	4000	76	66.64	45	4476797	SAMN08742775	2014	Brazil	sputum from CF
GCA_003062325.1	16140	*S. maltophilia*	3999	69	66.66	153	4472111	SAMN08744681	2013	Brazil	sputum
GCA_003062365.2	15180	*S. maltophilia*	4002	76	66.64	52	4474562	SAMN08767386	2012	Brazil	sputum
GCA_003062375.2	16185	*S. maltophilia*	4004	75	66.64	49	4477086	SAMN08767397	2013	Brazil	sputum
GCA_003205835.1	SJTL3	Genospecies 12	4367	77	66.27	1	4891004	SAMN09355365	2017	China	wastewater
GCA_003244375.1	SM-7180	*S. maltophilia*	4070	71	66.35	164	4558686	SAMN04978483	2012	India	respiratory
GCA_003244415.1	SM-2546	*S. maltophilia*	4188	76	66.48	145	4622246	SAMN04978487	2013	India	respiratory
GCA_003244425.1	SM-20065	*S. maltophilia*	4038	70	66.52	137	4502998	SAMN04978480	2012	India	blood
GCA_003244495.1	SM-17711	*S. sepilia*	3881	73	66.90	143	4333739	SAMN04978493	2012	India	blood
GCA_003244505.1	SM-6957	*S. sepilia*	3879	64	66.78	128	4300032	SAMN04978495	2013	India	blood
GCA_003244545.1	SM-10507	*S. muris*	4371	66	66.40	167	4783577	SAMN04978502	2012	India	cerebrospinal fluid
GCA_003244555.1	SM-16360	*S. muris*	4244	69	66.63	116	4711957	SAMN04978503	2012	India	blood
GCA_003244595.1	SM-38795	*P. hibiscicola*	3794	71	66.59	136	4227084	SAMN04978505	2013	India	blood
GCA_003244615.1	SM-3123	Genospecies 6	3560	70	66.92	114	4018217	SAMN04978506	2010	India	respiratory
GCA_003244625.1	SM-3226	*S. maltophilia*	4001	75	66.61	181	4485068	SAMN04978481	2012	India	blood
GCA_003244635.1	SM-325416	*S. maltophilia*	4035	73	66.60	166	4499696	SAMN04978482	2013	India	blood
GCA_003244645.1	SM-7882	*S. maltophilia*	3991	68	66.50	143	4404454	SAMN04978484	2012	India	respiratory
GCA_003244695.1	SM-480	*S. maltophilia*	3824	63	66.60	143	4293836	SAMN04978485	2013	India	respiratory
GCA_003244715.1	SM-19467	*S. maltophilia*	4175	73	66.53	162	4590278	SAMN04978490	2012	India	blood
GCA_003244725.1	SM-11522	*S. maltophilia*	4314	66	66.14	163	4771977	SAMN04978486	2012	India	blood

GCA_003244735.1	SM-30540	Genospecies 3	4098	69	66.64	147	4544008	SAMN04978491	2013	India	blood
GCA_003244785.1	SM-24179	*S. sepilia*	3819	77	66.69	140	4281516	SAMN04978494	2012	India	blood
GCA_003244795.1	SM-1006	*S. sepilia*	3894	69	66.63	119	4308028	SAMN04978499	2013	India	blood
GCA_003244805.1	SM-13670	*S. sepilia*	3851	73	66.59	136	4315911	SAMN04978497	2012	India	blood
GCA_003244855.1	SM-3112	*S. sepilia*	3847	74	66.65	150	4272080	SAMN04978500	2012	India	respiratory
GCA_003244865.1	SM-1389	*P. hibiscicola*	3926	76	66.56	122	4350701	SAMN04978504	2010	India	blood
GCA_003385015.1	AU32848	*S. maltophilia*	3911	55	66.64	155	4383122	SAMN09755211	2015	USA	sputum
GCA_003385135.1	AU30115	*S. maltophilia*	4166	72	66.42	89	4628959	SAMN09755198	2014	USA	sputum
GCA_003751565.1	BIGb0219	*S. chelatiphaga*	3517	66	66.88	16	4040198	SAMN10361619	unknown	unknown	unknown
GCA_003812985.1	FDAARGOS_507	*S. maltophilia*	4149	80	66.55	1	4577568	SAMN10163201	2015	USA	sputum
GCA_003935155.1	SM_218	*S. geniculata*	4072	66	66.43	76	4553216	SAMN10249184	2016	Pakistan	washroom sink
GCA_003935255.1	SM_055	*S. geniculata*	4006	68	66.48	51	4504029	SAMN10249176	2016	Pakistan	washroom sink
GCA_003935325.1	SM_047	Genospecies 1	4404	62	66.15	148	4861545	SAMN10249208	2016	Pakistan	washroom sink
GCA_003935335.1	SM_031	*S. geniculata*	4070	66	66.45	56	4552877	SAMN10249221	2016	Pakistan	washroom sink
GCA_003935615.1	SM_069	Genospecies 21	4418	69	66.10	72	4905593	SAMN10249185	2016	Pakistan	washroom sink
GCA_003935735.1	SM_220	*S. geniculata*	4071	67	66.43	70	4549704	SAMN10248948	2016	Pakistan	alcohol foam dispenser
GCA_003935825.1	SM_163	*S. geniculata*	4071	65	66.42	38	4552799	SAMN10249154	2016	Pakistan	washroom sink
GCA_003938995.1	FDAARGOS_508	*S. maltophilia*	4398	77	66.34	1	4807339	SAMN10163202	2015	USA	Throat (pharynx)
GCA_003970865.1	MDMC339	Genospecies 20	4271	71	66.14	185	4788525	SAMN10589628	2002	Morocco	desert sand
GCA_004346945.1	SVIA2	*S. maltophilia*	4023	77	66.63	1	4499711	SAMN09724453	2016	Mexico	soil
GCA_004522355.1	STN-AUH1	*P. hibiscicola*	3982	73	66.54	70	4412546	SAMN10819206	2018	Lebanon	Bacteria
GCA_004522395.1	STN-AUH2	*S. sepilia*	3970	72	66.70	94	4459062	SAMN10819528	2018	Lebanon	Bacteria
GCA_004522405.1	STN-AUH3	*P. hibiscicola*	3985	74	66.49	118	4417198	SAMN10819904	2018	Lebanon	Bacteria

GCA_004522445.1	STN-AUH5	*P. hibiscicola*	3987	74	66.48	129	4428145	SAMN10819978	2018	Lebanon	Bacteria
GCA_004522525.1	STN-AUH4	*S. sepilia*	3972	72	66.66	72	4461864	SAMN10819966	2018	Lebanon	Bacteria
GCA_004684085.1	D5.1	Genospecies 16	4189	67	66.04	91	4626840	SAMN11282838	2016	Australia	unknown
GCA_004793415.1	SMYN41	Genospecies 11	4480	69	66.60	50	4897188	SAMN11289620	2017	China	urine
GCA_004794075.1	NM62_B4-13	*S. nematodicola*	3776	71	67.60	42	4266549	SAMN10878308	2016	Canada	gut（ colon and cecum）
GCA_004920755.1	RC09	Genospecies 9	4079	73	66.15	112	4556223	SAMN08978033	2016	USA	Residence cold water tap
GCA_004920835.1	NEC1	Genospecies 9	4078	73	66.14	81	4559750	SAMN08978021	2016	USA	Hospital hot water tap
GCA_006120755.1	SMYN44	*S. maltophilia*	4177	68	66.31	56	4666192	SAMN11289624	2017	China	urine
GCA_006120765.1	SMYN42	*S. sepilia*	3890	67	66.72	14	4371528	SAMN11289619	2017	China	urine
GCA_006120775.1	SMYN45	*S. maltophilia*	4050	70	66.59	23	4545520	SAMN11289622	2017	China	sputum
GCA_006149095.1	SMYN43	*S. sepilia*	3889	68	66.72	12	4371843	SAMN11289621	2017	China	urine
GCA_006370585.1	476BLSM_MAD	*S. muris*	4436	75	66.44	64	4904652	SAMN12035360	2015	Madagascar	environmental
GCA_006385155.1	517SM	*S. muris*	4430	66	66.43	86	4892122	SAMN12057332	2015	Madagascar	Medical
GCA_006385195.1	646SM	*S. muris*	4434	69	66.43	65	4896286	SAMN12057334	2015	Madagascar	Medical
GCA_006385275.1	629SM	*S. muris*	4440	73	66.38	97	4913259	SAMN12057333	2015	Madagascar	Medical
GCA_006385415.1	PF-4	*S. indicatrix*	3888	72	66.43	26	4377836	SAMN11870792	2017	USA	Grower Feed
GCA_006385505.1	PF-8	*S. indicatrix*	4040	69	66.46	28	4520531	SAMN11870796	2017	USA	Grower Feed
GCA_006716455.1	1190	*S. muris*	3997	71	66.99	7	4436036	SAMN11512362	unknown	unknown	unknown
GCA_006970445.1	291	Genospecies 18	4063	71	66.72	2	4549249	SAMN10411010	2016	Brazil	rhizosphere

GCA_006974125.1	X28	Genospecies 2	4063	71	66.52	1	4554224	SAMN11032470	2017	China	aerobic granular sludge
GCA_007833655.1	SM 866	Genospecies 1	4692	74	66.03	1	5086181	SAMN09665072	2013	India	unknown
GCA_008502035.1	CC2	*S. pavanii*	4194	63	66.97	31	4644657	SAMN09862369	2014	Nigeria	unknown
GCA_008553765.1	ptp 12.25	Genospecies 1	4282	69	66.21	32	4765472	SAMN12586753	2017	Malaysia	ballast water
GCA_008693985.1	FDAARGOS_649	*S. maltophilia*	4158	78	66.53	1	4625884	SAMN11056364	unknown	USA	clinical isolate
GCA_009618035.1	KMM 349	Genospecies 10	4044	80	66.21	1	4578300	SAMD00190103	1991	Philippine Sea	deep-sea sponge
GCA_009676405.1	Sm53	*S. maltophilia*	4201	81	66.34	1	4682439	SAMN11652201	2018	Germany	unknown
GCA_009676425.1	sm454	*S. maltophilia*	4210	78	66.32	1	4685439	SAMN11652200	2018	Germany	unknown
GCA_009676445.1	sm-RA9	Genospecies 10	4474	83	65.63	1	5007582	SAMN11652199	2018	Germany	unknown
GCA_009676465.1	SKK55	*S. sepilia*	4206	77	66.25	1	4675446	SAMN11652198	2018	Germany	unknown
GCA_009676485.1	PEG-68	*S. muris*	4143	79	66.70	1	4635025	SAMN11652197	2018	Germany	unknown
GCA_009676505.1	PEG-390	*P. hibiscicola*	4119	78	66.44	1	4554024	SAMN11652195	2018	Germany	unknown
GCA_009676525.1	PEG-42	*S. africana*	4405	78	66.08	1	4854802	SAMN11652196	2018	Germany	unknown
GCA_009676545.1	PEG-305	*S. pavanii*	3986	76	67.20	1	4495508	SAMN11652194	2018	Germany	unknown
GCA_009676565.1	PEG-173	Genospecies 1	4238	77	66.11	1	4769515	SAMN11652193	2018	Germany	unknown
GCA_009676585.1	PEG-141	Genospecies 1	4605	84	66.13	1	5002295	SAMN11652192	2018	Germany	unknown
GCA_009676605.1	ICU331	*S. maltophilia*	4563	84	66.23	1	4995994	SAMN11652191	2018	Germany	unknown
GCA_010994075.1	STN-MAK7	*S. maltophilia*	4238	76	66.38	113	4736097	SAMN13247120	2019	Lebanon	unknown
GCA_010994225.1	STN-MAK8	*S. sepilia*	3878	74	66.81	193	4381617	SAMN13268959	2019	Lebanon	unknown
GCA_011067225.1	MER1	*S. lactitubi*	4047	76	66.00	1	4547296	SAMN14209924	2019	China	wastewater
GCA_011386925.1	NCTC10498	*S. maltophilia*	4509	78	66.25	1	4928653	SAMN14206046	2016	China	feces
GCA_012647025.1	NEB515	*S. maltophilia*	4354	78	66.43	1	4785515	SAMN14591332	unknown	unknown	unknown

GCA_012956725.1	steno33	*S. muris*	4014	72	67.03	76	4464622	SAMN14596987	2009	Brazil	catheter
GCA_012956765.1	steno32	*S. muris*	4012	72	67.03	91	4461798	SAMN14596986	2009	Brazil	catheter
GCA_012971765.1	CF13	*S. maltophilia*	4113	75	66.46	1	4591696	SAMN12500624	2011	Australia	sputum
GCA_013004645.1	ATCC 700475	*S. africana*	4134	74	66.33	29	4573818	SAMN14517832	unknown	Zaire	unknown
GCA_013184035.1	G1W4	*S. geniculata*	4144	72	66.33	59	4601319	SAMN14775808	2017	China	Sewage from swine farm
GCA_013184045.1	JHV2-2	*S. sepilia*	4006	76	66.53	59	4459755	SAMN14775815	2017	China	vegetable
GCA_013184055.1	G1W2	*S. geniculata*	4138	71	66.35	45	4596733	SAMN14775807	2017	China	Sewage from swine farm
GCA_013184065.1	FS40-1	*P. hibiscicola*	3879	75	66.56	44	4335129	SAMN14775810	2017	China	manure
GCA_013184135.1	JS56-1	*S. maltophilia*	3993	73	66.58	78	4525257	SAMN14775806	2017	China	caecum
GCA_013184165.1	G4S2	*S. sepilia*	3933	72	66.54	125	4415147	SAMN14775820	2017	China	Soil from swine farm
GCA_013184205.1	G4S2-1	*S. sepilia*	3924	72	66.58	82	4399953	SAMN14775819	2017	China	Soil from swine farm
GCA_013282585.1	NMBU_R2	*S. maltophilia*	4320	74	66.42	51	4765683	SAMN13865298	2018	Norway	surface water
GCA_013345715.1	SM130	*S. maltophilia*	4019	72	66.67	117	4478585	SAMN15177966	2015	Bulgaria	Urinary tract infection
GCA_013463455.1	OU152	Genospecies 10	4496	76	66.03	138	4968462	SAMN10131081	2012	Belgium	Oropharinx
GCA_013463465.1	PC255	*S. sepilia*	3836	71	66.70	62	4303968	SAMN10131080	2012	Germany	Perineum swab
GCA_013463485.1	OU111	Genospecies 6	3961	70	66.64	63	4441446	SAMN10131082	2012	Belgium	Oropharinx
GCA_013463495.1	OL16	*S. sepilia*	4142	68	66.49	75	4542287	SAMN10131078	2011	Slovenia	Oropharinx
GCA_013463535.1	PC257	*S. sepilia*	3838	71	66.68	73	4307850	SAMN10131079	2012	Germany	Perineum swab
GCA_013463565.1	OS87	*S. sepilia*	4417	69	66.30	105	4840771	SAMN10131076	2012	France	Oropharinx
GCA_013463585.1	OR41	*S. sepilia*	4253	69	66.35	86	4701856	SAMN10131077	2012	France	Oropharinx

GCA_013463595.1	OS91	*S. sepilia*	4410	72	66.29	141	4839728	SAMN10131075	2012	France	Oropharinx
GCA_013463605.1	PC274	*P. hibiscicola*	4009	65	66.21	53	4492579	SAMN10131074	2012	Germany	Perineum swab
GCA_013463655.1	S5720	*P. hibiscicola*	3939	69	66.52	58	4396510	SAMN10131073	2011	Spain	sputum
GCA_013463675.1	PU153	*S. muris*	4316	68	66.70	142	4762819	SAMN10131069	2012	Belgium	Perineum swab
GCA_013463685.1	S289	*S. sepilia*	4000	67	66.70	79	4510473	SAMN10131072	2011	Spain	sputum
GCA_013463715.1	OU141	*S. muris*	4363	66	66.41	172	4826140	SAMN10131070	2012	Belgium	Oropharinx
GCA_013463725.1	PU140	*S. muris*	4356	70	66.42	156	4827482	SAMN10131071	2012	Belgium	Perineum swab
GCA_013463755.1	PC240	*S. muris*	4460	69	66.69	128	4847867	SAMN10131068	2012	Germany	Perineum swab
GCA_013463775.1	PC232	Genospecies 7	4409	65	66.15	86	4825217	SAMN10131066	2012	Germany	Perineum swab
GCA_013463785.1	PC226	Genospecies 7	4410	66	66.15	86	4824438	SAMN10131065	2012	Germany	Perineum swab
GCA_013463795.1	PR45	*S. muris*	4014	66	66.96	66	4449230	SAMN10131067	2012	France	Perineum swab
GCA_013463805.1	EV5520	Genospecies 12	4695	69	66.25	137	5089844	SAMN10131064	2011	Spain	Environmental control
GCA_013463855.1	OU110	Genospecies 12	4593	69	66.10	87	5071543	SAMN10131063	2012	Belgium	Oropharinx
GCA_013463875.1	PU126	Genospecies 1	4452	62	66.11	105	4885645	SAMN10131059	2012	Belgium	Perineum swab
GCA_013463885.1	PU109	Genospecies 12	4598	68	66.10	108	5071214	SAMN10131062	2012	Belgium	Perineum swab
GCA_013463895.1	P815	Genospecies 1	4594	67	66.09	132	5005218	SAMN10131061	2005	Spain	Pharyngeal exudate
GCA_013463915.1	H5726	Genospecies 1	4427	64	66.08	139	4908212	SAMN10131060	2011	Spain	Bronchial aspirate
GCA_013463955.1	PC186	*S. geniculata*	4204	73	66.35	43	4676306	SAMN10131058	2012	Germany	Perineum swab
GCA_013463975.1	PC187	*S. geniculata*	4204	69	66.32	41	4679598	SAMN10131057	2012	Germany	Perineum swab
GCA_013463995.1	OC197	*S. geniculata*	4204	70	66.35	37	4675475	SAMN10131056	2012	Germany	Oropharinx
GCA_013464005.1	PU102	*S. africana*	4121	73	66.23	78	4604057	SAMN10131054	2012	Belgium	Oropharinx
GCA_013464035.1	PU101	*S. africana*	4163	72	66.17	68	4658515	SAMN10131055	2012	Belgium	Perineum swab
GCA_013464045.1	OU148	*S. africana*	4119	69	66.23	72	4604315	SAMN10131053	2012	Belgium	Oropharinx

GCA_013464065.1	PC313	Genospecies 14	4133	74	66.66	133	4639595	SAMN10131052	2012	Germany	Perineum swab
GCA_013464095.1	PC312	Genospecies 14	4170	74	66.42	144	4682822	SAMN10131050	2012	Germany	Perineum swab
GCA_013464105.1	PC314	Genospecies 14	4133	72	66.66	165	4638155	SAMN10131051	2012	Germany	Perineum swab
GCA_013464125.1	OG156	Genospecies 3	4353	68	66.19	68	4795669	SAMN10131049	2012	Slovenia	Oropharinx
GCA_013464155.1	OC323	*S. maltophilia*	4346	69	66.16	102	4843502	SAMN10131048	2012	Germany	Oropharinx
GCA_013464165.1	OU343	*S. maltophilia*	4238	67	66.36	84	4699051	SAMN10131047	2012	Belgium	Oropharinx
GCA_013464195.1	OL13	*S. maltophilia*	4346	62	66.47	129	4743353	SAMN10131044	2011	Slovenia	Oropharinx
GCA_013464205.1	OR79	*S. maltophilia*	4210	72	66.37	126	4693435	SAMN10131045	2012	France	Oropharinx
GCA_013464215.1	P626	*S. maltophilia*	4144	68	66.49	106	4590928	SAMN10131046	2004	Spain	Pharyngeal exudate
GCA_013464255.1	B5742	*S. maltophilia*	4292	69	66.20	129	4758630	SAMN10131042	2011	Spain	Bronchial aspirate
GCA_013464265.1	PC245	*S. maltophilia*	4352	65	66.23	131	4849798	SAMN10131043	2012	Germany	Perineum swab
GCA_013464295.1	S3149	*S. maltophilia*	3958	66	66.65	111	4449821	SAMN10131041	2007	Spain	Surgical wound
GCA_013464305.1	PL20	*S. maltophilia*	4319	72	66.37	102	4779000	SAMN10131040	2011	Slovenia	Perineum swab
GCA_013464315.1	OL11	*S. maltophilia*	4512	73	66.05	135	4956580	SAMN10131039	2011	Slovenia	Oropharinx
GCA_013464355.1	PL12	*S. maltophilia*	4503	70	66.05	108	4953483	SAMN10131038	2011	Slovenia	Perineum swab
GCA_013464365.1	B285	*S. maltophilia*	4169	70	66.38	124	4639329	SAMN10131037	2011	Spain	Bronchial aspirate
GCA_013464395.1	B5565	*S. maltophilia*	4392	67	66.27	151	4868054	SAMN10131036	2011	Spain	Bronchial aspirate
GCA_013464405.1	OU353	*S. maltophilia*	4299	67	66.44	153	4731077	SAMN10131035	2012	Belgium	Oropharinx
GCA_013464415.1	PU100	*S. maltophilia*	4274	68	66.50	135	4696073	SAMN10131034	2012	Belgium	Perineum swab
GCA_013464455.1	4834-R	Genospecies 12	4667	72	66.06	117	5151933	SAMN10131033	1999	Spain	clinical isolate
GCA_013464475.1	OC194	*S. geniculata*	4202	71	66.35	45	4674464	SAMN10131029	2012	Germany	Oropharinx
GCA_013464495.1	L9-5R5	Genospecies 13	4325	70	66.61	118	4851575	SAMN10131032	1999	Spain	clinical isolate

GCA_013464525.1	E50	*S. maltophilia*	4232	66	66.35	120	4685118	SAMN10131031	2009	Spain	sputum
GCA_013464555.1	PG157	*S. maltophilia*	4342	68	66.47	127	4761466	SAMN10131028	2012	Slovenia	Perineum swab
GCA_013464575.1	UV74	*S. muris*	4475	65	66.66	109	4881340	SAMN10131026	2009	Spain	vascular ulcer
GCA_013464585.1	M30	*S. muris*	4483	64	66.39	107	4892864	SAMN10131027	2009	Spain	decubitus ulcer
GCA_013464595.1	PC254	*S. sepilia*	3837	70	66.68	71	4308555	SAMN10131170	2012	Germany	Perineum swab
GCA_013464605.1	PC256	*S. sepilia*	3839	67	66.69	68	4307992	SAMN10131169	2012	Germany	Perineum swab
GCA_013464645.1	PC273	*P. hibiscicola*	3986	66	66.36	68	4468604	SAMN10131168	2012	Germany	Perineum swab
GCA_013464675.1	PC272	*P. hibiscicola*	3996	65	66.19	75	4490871	SAMN10131167	2012	Germany	Perineum swab
GCA_013464685.1	PC271	*P. hibiscicola*	3978	65	66.45	66	4453534	SAMN10131166	2012	Germany	Perineum swab
GCA_013464695.1	PC238	*S. muris*	4449	69	66.68	128	4845877	SAMN10131164	2012	Germany	Perineum swab
GCA_013464715.1	PC239	*S. muris*	4453	68	66.69	120	4849826	SAMN10131165	2012	Germany	Perineum swab
GCA_013464725.1	PC237	*S. muris*	4446	70	66.68	128	4851176	SAMN10131163	2012	Germany	Perineum swab
GCA_013464775.1	PC230	Genospecies 7	4410	69	66.15	86	4826267	SAMN10131161	2012	Germany	Perineum swab
GCA_013464785.1	PC231	Genospecies 7	4410	68	66.15	82	4826390	SAMN10131162	2012	Germany	Perineum swab
GCA_013464795.1	PC228	Genospecies 7	4414	71	66.15	93	4825073	SAMN10131159	2012	Germany	Perineum swab
GCA_013464805.1	PC227	Genospecies 7	4408	68	66.15	89	4824651	SAMN10131158	2012	Germany	Perineum swab
GCA_013464815.1	PC229	Genospecies 7	4414	68	66.15	97	4824711	SAMN10131160	2012	Germany	Perineum swab
GCA_013464875.1	OC195	*S. geniculata*	4206	71	66.35	44	4675638	SAMN10131154	2012	Germany	Oropharinx
GCA_013464885.1	PC185	*S. geniculata*	4202	72	66.35	32	4676816	SAMN10131157	2012	Germany	Perineum swab
GCA_013464895.1	OC196	*S. geniculata*	4204	74	66.35	47	4675992	SAMN10131155	2012	Germany	Oropharinx
GCA_013464905.1	PC184	*S. geniculata*	4208	72	66.35	39	4676328	SAMN10131156	2012	Germany	Perineum swab
GCA_013464915.1	PC315	Genospecies 14	4135	76	66.66	114	4646463	SAMN10131153	2012	Germany	Perineum swab
GCA_013464975.1	PC246	*S. maltophilia*	4354	65	66.24	148	4848460	SAMN10131151	2012	Germany	Perineum swab
GCA_013465025.1	OC322	*S. maltophilia*	4349	70	66.16	134	4840801	SAMN10131152	2012	Germany	Oropharinx
GCA_014076535.1	T50-20	Genospecies 1	4274	77	66.05	1	4777699	SAMN12671355	2018	China	biofilm reactor
GCA_014156975.1	WP1-W18-C	*S. maltophilia*	4416	82	66.22	1	4923943	SAMD00194327	2018	Japan	wastewater

	RE-01										
GCA_014171555.1	SoD9b	*S. sepilia*	4297	78	66.75	1	4415649	SAMN14081051	2014	Antarctica	Soil
GCA_014200115.1	SEMIA 4027	*S. maltophilia*	4035	72	66.63	32	4486072	SAMN13165421	unknown	unknown	unknown
GCA_014235265.1	NCTC10498	*S. maltophilia*	4083	63	66.43	44	4577239	SAMN15689527	2017	USA	Urinary tract infection
GCA_014236005.1	UHH_PEG 13-68-68	*S. maltophilia*	3993	72	66.65	1	4483650	SAMN15577962	2013	Germany	respiratory tract
GCA_014236025.1	UHH_PC240	*S. muris*	4459	70	66.69	1	4854229	SAMN15577961	2012	Germany	perineum
GCA_014236045.1	UHH_PC239	*S. muris*	4459	70	66.69	1	4855254	SAMN15577960	2012	Germany	perineum
GCA_014236085.1	UHH_SKK55	*S. sepilia*	4193	70	66.30	1	4666115	SAMN15577958	2018	Germany	respiratory tract
GCA_014236125.1	UHH_454	*S. maltophilia*	4170	71	66.38	1	4652546	SAMN15577956	2018	Germany	sputum
GCA_014398525.1	N0320	*S. maltophilia*	4356	77	66.35	79	4814846	SAMN14601825	2020	USA	nano-particles
GCA_014489825.1	1064209	*S. maltophilia*	3965	73	66.64	41	4446452	SAMN14089768	2018	USA	pulmonary
GCA_014489875.1	934728	*S. pavanii*	4074	71	67.11	84	4559093	SAMN14089766	2016	USA	pulmonary
GCA_014489885.1	1027369	*S. pavanii*	4100	70	67.16	86	4570612	SAMN14089767	2017	Italy	pulmonary
GCA_014581695.1	JZ56-1	*S. sepilia*	4037	75	66.50	99	4520851	SAMN14775818	2017	China	anal swab
GCA_014581725.1	JZ42	*S. sepilia*	3929	72	66.59	21	4385782	SAMN14775817	2017	China	anal swab
GCA_014581755.1	G1P11	*S. sepilia*	3874	71	66.79	10	4307168	SAMN14775814	2017	China	manure
GCA_014581765.1	GQ204	*S. pavanii*	3418	51	67.02	8	3797885	SAMN14775812	2017	China	anal swab
GCA_014581775.1	JZ50	*S. sepilia*	3951	72	66.55	66	4420963	SAMN14775816	2017	China	anal swab
GCA_014581825.1	HZ34	*P. hibiscicola*	3965	74	66.55	46	4413927	SAMN14775811	2017	China	nose swab

GCA_014581855.1	GQ250	*S. maltophilia*	4315	76	66.26	27	4791584	SAMN14775805	2017	China	anal swab
GCA_014581865.1	FS38	*P. hibiscicola*	3884	72	66.55	6	4302212	SAMN14775809	2017	China	manure
GCA_014581875.1	JZ104	*S. maltophilia*	3987	76	66.64	67	4486571	SAMN14775804	2017	China	anal swab
GCA_015956405.1	46-3	Genospecies 11	4138	78	66.64	25	4599487	SAMN10394026	2012	China	soil
GCA_015996605.1	STEN00200	*S. maltophilia*	4289	71	66.42	104	4716382	SAMN13622310	2018	USA	sputum
GCA_015996625.1	STEN00198	*S. maltophilia*	4110	74	66.53	162	4579508	SAMN13622308	2018	USA	wound
GCA_015996665.1	STEN00199	*S. maltophilia*	4039	74	66.68	154	4483911	SAMN13622309	2018	USA	sputum
GCA_015996685.1	STEN00197	*S. maltophilia*	4012	71	66.64	128	4472934	SAMN13622307	2018	USA	bronch
GCA_015996705.1	STEN00195	*S. muris*	4004	70	67.01	142	4430771	SAMN13622305	2018	USA	wound
GCA_015996715.1	STEN00190	*S. pavanii*	4154	71	67.13	108	4599104	SAMN13622304	2018	USA	sputum
GCA_015996735.1	STEN00196	*S. maltophilia*	4045	75	66.68	127	4485365	SAMN13622306	2018	USA	sputum
GCA_015996765.1	STEN00188	Genospecies 3	4420	76	66.09	137	4884038	SAMN13622302	2018	USA	sputum
GCA_015996785.1	STEN00189	*S. maltophilia*	4548	73	65.97	161	4936487	SAMN13622303	2018	USA	sputum
GCA_015996795.1	STEN00186	*S. maltophilia*	4301	72	66.25	195	4757644	SAMN13622300	2018	USA	sputum
GCA_015996805.1	STEN00187	*S. maltophilia*	4260	73	66.31	193	4714658	SAMN13622301	2018	USA	sputum
GCA_015996825.1	STEN00185	*S. geniculata*	3968	72	66.49	124	4433316	SAMN13622299	2018	USA	sputum
GCA_015996865.1	STEN00183	*S. maltophilia*	4344	80	66.27	154	4774139	SAMN13622297	2018	USA	sputum
GCA_015996895.1	STEN00181	*S. muris*	4237	71	66.73	134	4680748	SAMN13622295	2018	USA	sputum
GCA_015996915.1	STEN00180	*S. maltophilia*	4277	72	66.42	150	4709134	SAMN13622294	2018	USA	sputum
GCA_015996945.1	STEN00182	Genospecies 19	4492	71	66.35	188	4928495	SAMN13622296	2018	USA	sputum
GCA_015996965.1	STEN00177	*S. maltophilia*	4304	76	66.35	161	4755381	SAMN13622291	2018	USA	urine
GCA_015996985.1	STEN00179	*P. hibiscicola*	3815	73	66.58	121	4291830	SAMN13622293	2018	USA	blood
GCA_015996995.1	STEN00178	*S. maltophilia*	4323	75	66.39	167	4747860	SAMN13622292	2018	USA	sputum
GCA_015997005.1	STEN00175	*S. maltophilia*	4377	74	65.99	132	4832290	SAMN13622289	2018	USA	sputum
GCA_015997045.1	STEN00176	*S. geniculata*	4187	72	66.28	97	4642250	SAMN13622290	2018	USA	wound
GCA_015997065.1	STEN00173	Genospecies 13	4552	75	66.52	164	5006193	SAMN13622287	2018	USA	wound

GCA_015997075.1	STEN00174	*S. geniculata*	4323	71	66.13	104	4774857	SAMN13622288	2018	USA	wound
GCA_015997095.1	STEN00170	Genospecies 10	4450	77	65.86	182	4946539	SAMN13622284	2018	USA	sputum
GCA_015997125.1	STEN00172	*S. maltophilia*	4021	74	66.68	150	4467119	SAMN13622286	2018	USA	sputum
GCA_015997135.1	STEN00171	*S. maltophilia*	3948	74	66.70	129	4394438	SAMN13622285	2018	USA	sputum
GCA_015997165.1	STEN00167	Genospecies 2	4268	72	66.25	124	4800492	SAMN13622281	2018	USA	wound
GCA_015997195.1	STEN00169	*S. geniculata*	4304	73	66.27	77	4731164	SAMN13622283	2018	USA	blood
GCA_015997205.1	STEN00168	*S. muris*	4074	73	66.80	115	4574267	SAMN13622282	2018	USA	sputum
GCA_015997285.1	STEN00159	*S. muris*	4287	72	66.58	25	4727299	SAMN11285294	2018	USA	blood
GCA_015997295.1	STEN00160	*S. muris*	4580	74	66.69	81	4911532	SAMN11285295	2018	USA	sputum
GCA_015997305.1	STEN00155	*S. maltophilia*	4065	73	66.60	35	4532961	SAMN10437258	2018	USA	sputum
GCA_015997345.1	STEN00158	*S. pavanii*	4147	72	67.09	34	4627717	SAMN11285293	2018	USA	bronch
GCA_015997385.1	STEN00152	*S. muris*	4222	73	66.59	17	4662818	SAMN11285292	2018	USA	bronch
GCA_015997395.1	STEN00151	*S. maltophilia*	4398	76	66.34	60	4809749	SAMN10437257	2018	USA	sputum
GCA_015997415.1	STEN00149	Genospecies 11	4362	74	66.57	29	4829694	SAMN10437256	2018	USA	blood
GCA_015997445.1	STEN00148	*S. muris*	4289	73	66.57	27	4736868	SAMN10437255	2018	USA	sputum
GCA_015997455.1	STEN00147	*S. pavanii*	3928	71	67.22	66	4321756	SAMN11285291	2018	USA	sputum
GCA_015997485.1	STEN00146	Genospecies 13	4352	75	66.59	38	4831466	SAMN11285290	2018	USA	sputum
GCA_015997495.1	STEN00145	Genospecies 12	4651	73	66.16	39	5080735	SAMN11285289	2018	USA	sputum
GCA_015997525.1	STEN00144	*S. maltophilia*	4448	72	66.18	25	4852184	SAMN11285288	2018	USA	sputum
GCA_015997535.1	STEN00141	*S. maltophilia*	4420	77	66.31	57	4839601	SAMN11285285	2018	USA	urine
GCA_015997565.1	STEN00142	*S. muris*	4420	73	66.58	40	4831228	SAMN11285286	2018	USA	sputum
GCA_015997575.1	STEN00140	*S. muris*	4186	73	66.69	56	4672606	SAMN10437254	2018	USA	sputum
GCA_015997605.1	STEN00143	*S. africana*	4293	73	66.17	39	4744306	SAMN11285287	2018	USA	sputum
GCA_015997615.1	STEN00139	*S. maltophilia*	4414	74	65.98	26	4863291	SAMN11285284	2018	USA	sputum
GCA_015997665.1	STEN00099	*S. muris*	4456	74	66.55	85	4917530	SAMN11285279	2018	USA	bronch
GCA_015997705.1	STEN00136	Genospecies 1	4395	70	66.00	191	4900371	SAMN11285281	2018	USA	sputum

GCA_015997715.1	STEN00100	*S. maltophilia*	4204	69	66.45	106	4643621	SAMN11285280	2018	USA	sputum
GCA_015997745.1	STEN00098	*S. maltophilia*	4403	76	66.27	74	4836825	SAMN10437253	2018	USA	sputum
GCA_015997755.1	STEN00096	*S. sepilia*	3812	71	66.67	27	4269371	SAMN11285277	2018	USA	sputum
GCA_015997785.1	STEN00091	Genospecies 1	4419	73	66.21	120	4907207	SAMN10437251	2018	USA	sputum
GCA_015997795.1	STEN00097	*S. maltophilia*	4028	74	66.68	35	4484654	SAMN11285278	2018	USA	sputum
GCA_015997805.1	STEN00092	*S. geniculata*	4054	70	66.41	149	4523993	SAMN11285276	2018	USA	wound
GCA_015997845.1	STEN00095	*S. maltophilia*	4491	77	66.24	63	4924988	SAMN10437252	2018	USA	sputum
GCA_015997855.1	STEN00090	*S. maltophilia*	4307	74	66.46	180	4784766	SAMN10437250	2018	USA	sputum
GCA_015997885.1	STEN00089	*S. geniculata*	4413	72	66.36	87	4779200	SAMN10437249	2018	USA	sputum
GCA_015997895.1	STEN00088	*S. pavanii*	3998	72	67.33	95	4486563	SAMN10437248	2018	USA	sputum
GCA_015997905.1	STEN00087	*S. maltophilia*	4468	75	66.39	174	4923322	SAMN10437247	2018	USA	sputum
GCA_015997945.1	STEN00086	*S. maltophilia*	4304	73	66.39	182	4799820	SAMN10437246	2018	USA	unknown
GCA_015997995.1	STEN00080	Genospecies 7	4306	74	66.25	98	4756835	SAMN10437240	2018	USA	sputum
GCA_015998215.1	STEN00065	*S. muris*	4369	73	66.56	174	4834754	SAMN10437227	2017	USA	unknown
GCA_015998285.1	STEN00062	*S. maltophilia*	4002	73	66.36	82	4426045	SAMN10437224	2017	USA	sputum
GCA_015998295.1	STEN00063	*S. muris*	3979	71	66.99	169	4420836	SAMN10437225	2017	USA	sputum
GCA_015998305.1	STEN00064	Genospecies 7	4459	75	66.11	134	4909911	SAMN10437226	2017	USA	wound
GCA_015998335.1	STEN00061	*S. maltophilia*	4322	80	66.40	3	4768750	SAMN10437223	2017	USA	sputum
GCA_015998355.1	STEN00060	*S. maltophilia*	4260	74	66.55	180	4697829	SAMN10437222	2017	USA	sputum
GCA_015998385.1	STEN00058	*S. maltophilia*	4371	75	66.36	128	4853489	SAMN10437220	2017	USA	sputum
GCA_015998395.1	STEN00059	Genospecies 3	4422	77	66.26	155	4891585	SAMN10437221	2017	USA	blood
GCA_015998415.1	STEN00057	*S. maltophilia*	4315	73	66.32	158	4802734	SAMN10437219	2017	USA	wound
GCA_015998455.1	STEN00055	*S. maltophilia*	4503	75	66.26	174	4931396	SAMN10437217	2017	USA	wound
GCA_015998495.1	STEN00053	*S. muris*	4428	73	66.61	37	4817494	SAMN10437215	2017	USA	sputum
GCA_015998525.1	STEN00052	Genospecies 7	4410	74	66.17	62	4839447	SAMN10437214	2017	USA	sputum
GCA_015998545.1	STEN00051	*S. maltophilia*	4329	80	66.39	2	4773645	SAMN10437213	2017	USA	sputum

GCA_015998565.1	STEN00050	*S. muris*	4535	74	66.64	93	4924826	SAMN10437212	2017	USA	sputum
GCA_015998575.1	STEN00049	Genospecies 13	4294	76	66.71	59	4783202	SAMN10437211	2017	USA	sputum
GCA_015998585.1	STEN00048	*S. maltophilia*	4447	75	66.29	90	4879494	SAMN10437210	2017	USA	sputum
GCA_015998605.1	STEN00047	*S. maltophilia*	4416	71	66.14	74	4917506	SAMN10437209	2017	USA	wound
GCA_015998645.1	STEN00046	*S. geniculata*	3956	73	66.59	142	4443889	SAMN10437208	2017	USA	sputum
GCA_015998695.1	STEN00042	*S. maltophilia*	4568	74	66.16	175	4996731	SAMN10437204	2017	USA	sputum
GCA_015998765.1	STEN00037	*S. maltophilia*	4566	74	66.13	143	4994131	SAMN10437201	2017	USA	sputum
GCA_015998775.1	STEN00036	Genospecies 6	3813	73	66.92	44	4220585	SAMN10437200	2017	USA	wound
GCA_015998785.1	STEN00035	Genospecies 1	4482	71	65.98	109	4984653	SAMN10437199	2017	USA	scope
GCA_015998795.1	STEN00038	*S. muris*	4454	72	66.65	114	4839583	SAMN10437202	2017	USA	sputum
GCA_015998845.1	STEN00034	*S. muris*	4344	72	66.68	122	4797974	SAMN10437198	2017	USA	urine
GCA_015998865.1	STEN00033	*S. geniculata*	4125	71	66.44	81	4579848	SAMN10437197	2017	USA	sputum
GCA_015998875.1	STEN00032	*S. geniculata*	4547	73	66.16	103	4949175	SAMN10437196	2017	USA	wound
GCA_015998885.1	STEN00031	*S. muris*	4273	73	66.66	140	4725433	SAMN10437195	2017	USA	sputum
GCA_015998895.1	STEN00030	Genospecies 3	4369	75	66.36	108	4833543	SAMN10437194	2017	USA	sputum
GCA_015998945.1	STEN00028	*S. muris*	4152	73	66.80	107	4637700	SAMN10437193	2017	USA	sputum
GCA_015998965.1	STEN00025	Genospecies 11	4249	77	66.75	131	4726361	SAMN10437191	2017	USA	sputum
GCA_015998985.1	STEN00021	*S. maltophilia*	4512	72	66.31	46	4947929	SAMN10437190	2017	USA	sputum
GCA_016082115.1	Oak Ridge strain 02	*S. africana*	4362	69	66.17	1	4815503	SAMN15249472	1988	USA	Nuclear waste
GCA_016126935.1	FDAARGOS_1044	*S. maltophilia*	4563	80	66.12	1	5004265	SAMN16357213	unknown	USA	unknown
GCA_016652805.1	STEN00020	*S. maltophilia*	4109	74	66.46	63	4589029	SAMN10437189	2017	USA	sputum
GCA_016659185.1	DHHJ	Genospecies 1	4210	79	66.33	1	4682909	SAMN17255814	2005	China	soil
GCA_016735555.1	AMOa6	*S. maltophilia*	3965	73	66.64	106	4437116	SAMN17278865	2019	China	unknown
GCA_017149635.1	STEN00266	Genospecies 6	3780	70	66.61	104	4212119	SAMN17965153	2019	USA	wound

GCA_017149735.1	STEN00260	*S. geniculata*	3970	70	66.60	137	4428354	SAMN17965148	2019	USA	sputum
GCA_017149835.1	STEN00257	*S. maltophilia*	4322	72	66.47	188	4727415	SAMN17965145	2019	USA	sputum
GCA_017149855.1	STEN00246	*S. africana*	4384	69	66.14	172	4827912	SAMN17965137	2019	USA	sputum
GCA_017149885.1	STEN00255	*S. africana*	4385	70	66.08	168	4853314	SAMN17965143	2019	USA	wound
GCA_017149915.1	STEN00244	*S. muris*	4079	71	66.89	169	4516465	SAMN17965135	2019	USA	wound
GCA_017149955.1	STEN00207	*S. maltophilia*	4144	72	66.49	184	4569970	SAMN17965099	2019	USA	sputum
GCA_017149995.1	STEN00231	*S. muris*	4140	71	66.68	154	4641887	SAMN17965122	2019	USA	sputum
GCA_017150035.1	STEN00229	*S. geniculata*	3938	73	66.59	134	4403040	SAMN17965120	2019	USA	sputum
GCA_017150135.1	STEN00243	*S. africana*	4093	72	66.27	158	4550455	SAMN17965134	2019	USA	sputum
GCA_017150255.1	STEN00236	*S. pavanii*	4284	69	67.03	180	4709865	SAMN17965127	2019	USA	sputum
GCA_017150275.1	STEN00238	*S. maltophilia*	4162	70	66.36	192	4585990	SAMN17965129	2019	USA	sputum
GCA_017150335.1	STEN00235	*S. lactitubi*	4026	69	66.32	129	4558573	SAMN17965126	2019	USA	sputum
GCA_017150355.1	STEN00209	*S. maltophilia*	4386	75	66.02	180	4836214	SAMN17965101	2018	USA	sputum
GCA_017150375.1	STEN00233	*S. maltophilia*	3996	74	66.67	174	4483495	SAMN17965124	2019	USA	sputum
GCA_017150415.1	STEN00208	Genospecies 13	4274	77	66.69	166	4782091	SAMN17965100	2018	USA	sputum
GCA_017150475.1	STEN00217	*S. geniculata*	4386	72	66.28	129	4801947	SAMN17965109	2019	USA	sputum
GCA_017150515.1	STEN00216	*S. geniculata*	4001	71	66.65	157	4482741	SAMN17965108	2019	USA	Tissue
GCA_017150555.1	STEN00222	*S. maltophilia*	4245	73	66.35	186	4717704	SAMN17965114	2019	USA	sputum
GCA_017150615.1	STEN00215	*S. geniculata*	4003	71	66.65	130	4474238	SAMN17965107	2019	USA	sputum
GCA_017150635.1	STEN00218	*S. pavanii*	4164	68	67.15	184	4580697	SAMN17965110	2019	USA	sputum
GCA_017150655.1	STEN00214	*S. maltophilia*	4268	73	66.42	165	4643274	SAMN17965106	2019	USA	sputum
GCA_017150675.1	STEN00212	Genospecies 1	4256	72	66.16	186	4743817	SAMN17965104	2018	USA	wound
GCA_017150685.1	STEN00213	*S. pavanii*	3896	68	67.40	187	4368328	SAMN17965105	2019	USA	sputum
GCA_017150755.1	STEN00206	*S. pavanii*	4213	75	66.93	139	4623608	SAMN17965098	2018	USA	sputum
GCA_017287455.1	15984	*S. pavanii*	3941	72	67.35	58	4424524	SAMN17860425	2017	Nigeria	ear swab
GCA_017287615.1	15982	*S. sepilia*	4014	72	66.48	72	4476389	SAMN17860424	2017	Nigeria	stool

GCA_017287815.1	15973	*P. hibiscicola*	3963	75	66.59	70	4409085	SAMN17860417	2017	Nigeria	urine
GCA_017287835.1	15972	*S. sepilia*	4018	72	66.49	56	4480690	SAMN17860416	2018	Nigeria	wound
GCA_017287995.1	15971	*S. sepilia*	4023	72	66.48	65	4483848	SAMN17860415	2018	Nigeria	wound
GCA_017292165.1	15950	*S. sepilia*	4055	72	66.48	71	4500852	SAMN17860398	2017	Nigeria	stool
GCA_017315435.1	S76-3	*P. hibiscicola*	3962	68	66.56	63	4413424	SAMN18099585	2020	USA	organic lettuce and spinach
GCA_017581785.1	16001	*S. sepilia*	4049	73	66.46	67	4503124	SAMN17860438	2018	Nigeria	urine
GCA_017582325.1	16022	*S. sepilia*	4021	72	66.49	63	4479014	SAMN17860450	2018	Nigeria	urine
GCA_017582525.1	15992	*S. sepilia*	4008	71	66.48	141	4468665	SAMN17860433	2017	Nigeria	urine
GCA_017582605.1	15989	*S. sepilia*	4017	72	66.49	70	4482344	SAMN17860430	2017	Nigeria	urine
GCA_017582615.1	15990	*S. sepilia*	4019	72	66.48	92	4475878	SAMN17860431	2017	Nigeria	urine
GCA_017582655.1	15987	*S. maltophilia*	4299	74	66.39	66	4741983	SAMN17860428	2017	Nigeria	urine
GCA_017838015.1	15977	*S. maltophilia*	4295	76	66.38	79	4740258	SAMN17860419	2017	Nigeria	urine
GCA_017838025.1	15967	*S. maltophilia*	4296	76	66.39	79	4742006	SAMN17860412	2018	Nigeria	urine
GCA_018917605.1	N82	*S. maltophilia*	4194	72	66.36	57	4695583	SAMN19374041	2019	China	feces
GCA_019048905.1	Col1	*S. geniculata*	3982	79	66.48	1	4458565	SAMN19803883	2018	South Korea	pavement soil
GCA_019203765.1	O1	*S. maltophilia*	5608	79	66.55	1	4516998	SAMN19929417	2020	South Korea	feces
GCA_019807975.1	UENF-4GII	*S. sepilia*	3957	74	66.55	3	4439904	SAMN15048632	2012	Brazil	vermicompost
GCA_019891375.1	6960	*S. maltophilia*	4282	72	66.22	81	4756179	SAMN21169357	2016	Serbia	unknown
GCA_019969825.1	ESA45A	*S. lactitubi*	4009	68	66.10	27	4527427	SAMN20394475	unknown	USA	soil1
GCA_020080085.1	XL133	*S. maltophilia*	4139	82	66.42	1	4630138	SAMN21404530	2018	China	cucumber rhizosphere
GCA_020132085.1	JZL8	*S. geniculata*	4174	77	66.32	1	4635432	SAMN15794714	2017	China	unknown
GCA_020166085.1	NAA11	*S. maltophilia*	4302	72	66.43	141	4731402	SAMN20525554	2009	Benin	maize soil

GCA_020551925.1	1	*S. geniculata*	3995	71	66.57	63	4449516	SAMN21467686	2017	Tunisia	rhizosphere roots
GCA_020641075.2	ACYCa.1J	*S. maltophilia*	4181	78	66.41	1	4670277	SAMN21163372	2020	China, Hong Kong	freshwater stream
GCA_020641085.2	ACYCd.9D	*S. sepilia*	3957	78	66.49	1	4435369	SAMN21163379	2020	China, Hong Kong	freshwater stream
GCA_020641095.2	ACYCc.3B	*S. muris*	3984	77	66.96	1	4416465	SAMN21163375	2020	China, Hong Kong	freshwater stream
GCA_020641355.2	ACYCe.8N	*S. maltophilia*	4154	77	66.32	3	4638222	SAMN21163378	2020	China, Hong Kong	freshwater stream
GCA_020641365.2	ACYCb.10K	*P. hibiscicola*	4188	78	66.21	1	4636985	SAMN21163380	2020	China, Hong Kong	freshwater stream
GCA_020641395.2	ACYCa.6E	*S. maltophilia*	4202	77	66.46	2	4646175	SAMN21163376	2020	China, Hong Kong	freshwater stream
GCA_020641415.2	ACYCb.1K	*P. hibiscicola*	3931	78	66.48	1	4410708	SAMN21163373	2020	China, Hong Kong	freshwater stream
GCA_020641425.2	ACYCb.6H	*P. hibiscicola*	3958	79	66.45	1	4417619	SAMN21163377	2020	China, Hong Kong	freshwater stream

GCA_020641455.2	ACYCa.2H	*S. maltophilia*	4110	81	66.51	1	4584972	SAMN21163374	2020	China, Hong Kong	freshwater stream
GCA_021117175.1	2013-SM24	*S. maltophilia*	4039	82	66.40	1	4533092	SAMN23441570	2013	China	drinking water
GCA_021117195.1	2013-SM15	*S. maltophilia*	4194	81	66.29	1	4702572	SAMN23441569	2013	China	drinking water
GCA_021117215.1	2013-SM13	*S. pavanii*	4115	79	66.99	1	4613698	SAMN23441568	2013	China	drinking water
GCA_021117235.1	2013-SM12	*S. pavanii*	4115	79	66.99	1	4612229	SAMN23441567	2013	China	drinking water
GCA_021117255.1	2013-SM4	*S. sepilia*	4114	76	66.35	1	4575852	SAMN23441566	2013	China	drinking water
GCA_021139765.1	KJ	*S. maltophilia*	4458	70	66.25	70	4879596	SAMN21353060	2003	China, Taiwan	sputum
GCA_021378335.1	ZT1	*P. hibiscicola*	3921	74	66.51	1	4391471	SAMN18275197	2019	China	bile acid
GCA_021441865.1	WGB211	Genospecies 7	4468	80	66.03	1	4913676	SAMN24492124	2021	China	shale
GCA_021554075.1	SM-5	*S. geniculata*	4118	71	66.44	9	4604394	SAMN13826194	2017	China	unknown
GCA_021554095.1	SM-2	*S. pavanii*	4164	74	67.05	22	4637774	SAMN13781871	2017	China	unknown
GCA_021554115.1	SM-1	*S. pavanii*	4165	74	67.05	22	4637291	SAMN13781462	2017	China	unknown
GCA_021554125.1	SM-3	*S. maltophilia*	4162	71	66.47	30	4630846	SAMN13812697	2017	China	unknown
GCA_021554135.1	SM-4	*S. maltophilia*	4160	73	66.48	29	4626169	SAMN13814255	2017	China	unknown
GCA_021554175.1	SM-7	*S. geniculata*	4089	71	66.38	12	4575607	SAMN13826448	2017	China	unknown
GCA_021554195.1	SM-6	*S. sepilia*	4133	73	66.36	28	4580179	SAMN13826216	2017	China	unknown
GCA_021554235.1	SM-9	*S. sepilia*	4299	77	66.23	32	4719212	SAMN13826468	2017	China	unknown
GCA_021554255.1	SM-8	*S. sepilia*	4069	71	66.46	12	4523053	SAMN13826465	2017	China	unknown
GCA_021554275.1	SM-12	*S. pavanii*	4314	73	66.98	31	4748719	SAMN13826472	2017	China	unknown
GCA_021554295.1	SM-15	*S. sepilia*	3990	73	66.56	23	4447722	SAMN13826476	2017	China	unknown
GCA_021554315.1	SM-16	*S. sepilia*	3985	73	66.57	12	4441114	SAMN13826882	2017	China	unknown
GCA_021554335.1	SM-17	*S. maltophilia*	4258	74	66.36	46	4741497	SAMN13826884	2017	China	unknown
GCA_021554355.1	SM-11	*S. maltophilia*	4263	75	66.42	42	4681805	SAMN13826883	2017	China	unknown

GCA_021554375.1	SM-18	*S. maltophilia*	4262	74	66.36	47	4742314	SAMN13826906	2017	China	unknown
GCA_021554385.1	SM-19	*S. sepilia*	3929	73	66.63	12	4387593	SAMN13826907	2017	China	unknown
GCA_021554415.1	SM-20	*S. sepilia*	3929	73	66.63	12	4387374	SAMN13826929	2017	China	unknown
GCA_021554435.1	SM-23	*S. sepilia*	3922	70	66.63	19	4384391	SAMN13830169	2017	China	unknown
GCA_021554455.1	SM-26	*S. muris*	4379	76	66.50	20	4829011	SAMN13830186	2017	China	unknown
GCA_021554475.1	SM-24	*S. sepilia*	3918	74	66.63	19	4385418	SAMN13830182	2017	China	unknown
GCA_021554495.1	SM-27	*S. sepilia*	4116	71	66.41	24	4577540	SAMN13830196	2017	China	unknown
GCA_021554505.1	SM-25	*S. sepilia*	3687	56	66.50	36	4074747	SAMN13830185	2017	China	unknown
GCA_021554535.1	SM-28	*S. sepilia*	4115	71	66.41	23	4577420	SAMN13830213	2017	China	unknown
GCA_021554555.1	SM-29	*S. maltophilia*	4287	74	66.32	26	4782584	SAMN13830222	2017	China	unknown
GCA_021554575.1	SM-31	*S. maltophilia*	4291	72	66.37	23	4758939	SAMN13830224	2017	China	unknown
GCA_021554595.1	SM-30	*S. maltophilia*	4291	73	66.36	43	4762722	SAMN13830223	2017	China	unknown
GCA_021554615.1	SM-32	*S. maltophilia*	4297	72	66.37	25	4759892	SAMN13830225	2017	China	unknown
GCA_022014735.1	PSKL2	*S. maltophilia*	4089	79	66.48	1	4574660	SAMN25416139	2021	China, Hong Kong	Storm drain water
GCA_022630595.1	JL-F-16	*S. muris*	4077	73	66.87	36	4518745	SAMN26589451	2016	China	unknown
GCA_022630615.1	JL-F-22-1	*S. africana*	4057	75	66.40	55	4503600	SAMN26589452	2016	China	unknown
GCA_022630625.1	JL-F-14	*S. maltophilia*	4309	77	66.53	69	4737912	SAMN26589450	2016	China	unknown
GCA_022630635.1	JL-F-26-1	*S. maltophilia*	4112	79	66.61	94	4587496	SAMN26589453	2016	China	unknown
GCA_022630715.1	JL-F-29	*S. maltophilia*	4121	74	66.65	96	4575161	SAMN26589454	2016	China	unknown
GCA_022630735.1	NMG-F-4	*S. maltophilia*	4142	77	66.39	83	4662270	SAMN26589457	2016	China	unknown
GCA_022630745.1	JL-F-51	*S. geniculata*	4026	71	66.50	33	4508054	SAMN26589456	2016	China	unknown
GCA_022630775.1	NMG-F-5	*S. geniculata*	4013	70	66.51	44	4516564	SAMN26589458	2016	China	unknown
GCA_022630795.1	NMG-F-9	*S. geniculata*	4097	72	66.51	15	4541996	SAMN26589459	2016	China	unknown
GCA_022630815.1	MNG-F-11	*S. maltophilia*	4146	77	66.37	66	4663651	SAMN26589460	2016	China	unknown

GCA_022630835.1	NMG-F-18-2	*S. geniculata*	4056	70	66.51	33	4532185	SAMN26589463	2016	China	unknown
GCA_022630845.1	NMG-F-18-1	*S. muris*	4016	72	67.06	48	4455330	SAMN26589462	2016	China	unknown
GCA_022630865.1	NMG-F-17	*S. maltophilia*	4145	76	66.40	72	4657304	SAMN26589461	2016	China	unknown
GCA_022630895.1	NMG-F-23	*S. geniculata*	3982	75	66.50	31	4479821	SAMN26589464	2016	China	unknown
GCA_022630915.1	NMG-F-30	*S. maltophilia*	4145	77	66.37	53	4659850	SAMN26589465	2016	China	unknown
GCA_022630935.1	NMG-F-34-1	*S. maltophilia*	4145	77	66.37	56	4659970	SAMN26589466	2016	China	unknown
GCA_022630955.1	NMG-F-36	*S. geniculata*	4064	70	66.46	73	4547605	SAMN26589468	2016	China	unknown
GCA_022630975.1	NMG-F-46	*S. geniculata*	3971	72	66.54	25	4449140	SAMN26589469	2016	China	unknown
GCA_022630995.1	NMG-F-49-2	*S. maltophilia*	4147	77	66.37	58	4662448	SAMN26589470	2016	China	unknown
GCA_022631015.1	NMG-F-52	*S. africana*	4014	71	66.37	40	4482664	SAMN26589472	2016	China	unknown
GCA_022631035.1	NMG-F-54	*S. geniculata*	4014	70	66.50	40	4516279	SAMN26589473	2016	China	unknown
GCA_022631075.1	NMG-F-34-2	*S. geniculata*	4017	70	66.51	43	4517091	SAMN26589467	2016	China	unknown
GCA_022631135.1	NMG-F-51	*S. geniculata*	4026	71	66.50	33	4508054	SAMN26589471	2016	China	unknown
GCA_022750675.1	258/3	*S. maltophilia*	4270	67	66.37	76	4741595	SAMN25182284	2020	Russia	unknown
GCA_022750685.1	41734/1	*S. maltophilia*	4083	69	66.54	97	4578465	SAMN25182285	2020	Russia	unknown
GCA_022750695.1	41761/4	*S. maltophilia*	4273	70	66.51	110	4675296	SAMN25182288	2020	Russia	unknown
GCA_022750715.1	41753/3	*S. sepilia*	4093	59	66.37	113	4537581	SAMN25182286	2020	Russia	unknown
GCA_022750755.1	41761/2	*S. maltophilia*	4275	75	66.46	110	4680678	SAMN25182287	2020	Russia	unknown
GCA_022750775.1	177/4	*S. maltophilia*	4165	64	66.34	116	4611231	SAMN25182280	2020	Russia	unknown
GCA_022750785.1	203/3	*S. muris*	4337	70	66.60	51	4787667	SAMN25182281	2020	Russia	unknown
GCA_022750795.1	163/1	*S. maltophilia*	4451	76	66.19	65	4857410	SAMN25182279	2020	Russia	unknown

GCA_022750835.1	210/3	*S. maltophilia*	4458	74	66.24	116	4875034	SAMN25182282	2020	Russia	unknown
GCA_022750845.1	213/4	*S. maltophilia*	4329	68	66.36	112	4783572	SAMN25182283	2020	Russia	unknown
GCA_022750875.1	159/1	*S. maltophilia*	4474	67	66.34	98	4902854	SAMN25182278	2020	Russia	unknown
GCA_022750885.1	149/1	*S. maltophilia*	4318	67	66.34	135	4782877	SAMN25182277	2020	Russia	unknown
GCA_022750915.1	144/2	*S. maltophilia*	3968	66	66.30	69	4444471	SAMN25182276	2020	Russia	unknown
GCA_022750935.1	17923/1	*S. maltophilia*	4314	61	66.36	96	4779095	SAMN25182274	2020	Russia	unknown
GCA_022750955.1	112/1	*S. maltophilia*	4211	75	66.42	57	4677932	SAMN25182275	2020	Russia	unknown
GCA_022750975.1	17917/2	*S. maltophilia*	4219	62	66.35	151	4679963	SAMN25182273	2020	Russia	unknown
GCA_022750995.1	320/2	*S. maltophilia*	3959	59	66.70	114	4444053	SAMN25182270	2020	Russia	unknown
GCA_022751035.1	320/1	*S. maltophilia*	3950	65	66.71	118	4441225	SAMN25182269	2020	Russia	unknown
GCA_022751045.1	452/1	*S. maltophilia*	4021	62	66.61	80	4480464	SAMN25182271	2020	Russia	unknown
GCA_022751075.1	92/2	*S. maltophilia*	4269	63	66.31	105	4755263	SAMN25182268	2020	Russia	unknown
GCA_022751095.1	55/3	*S. maltophilia*	4265	65	66.52	131	4702129	SAMN25182265	2020	Russia	unknown
GCA_022751115.1	37/4	*S. maltophilia*	4083	67	66.63	117	4490888	SAMN25182264	2020	Russia	unknown
GCA_022751135.1	84/1	*S. maltophilia*	4300	66	66.34	107	4775654	SAMN25182267	2020	Russia	unknown
GCA_022751145.1	60/4	Genospecies 2	4305	60	66.34	85	4790450	SAMN25182266	2020	Russia	unknown
GCA_022751175.1	24/2	*S. maltophilia*	4177	60	66.43	124	4626948	SAMN25182263	2020	Russia	unknown
GCA_023277525.1	FZD2	Genospecies 12	4312	80	66.37	1	4817124	SAMN20569879	2018	Poland	activated sludge
GCA_023702195.1	212	*S. maltophilia*	4602	73	66.29	157	4959053	SAMN28868844	2020	China	unknown
GCA_023702295.1	156	*P. hibiscicola*	3958	73	66.33	114	4427356	SAMN28868691	2018	China	unknown
GCA_023702495.1	142	*S. maltophilia*	4368	79	66.20	1	4830983	SAMN28867336	2018	China	unknown
GCA_023714625.1	FAIRING W8B-1	*S. muris*	4457	73	66.54	73	4898338	SAMN27921962	2013	USA	Cleanroom
GCA_024126675.1	S 297-4	Genospecies 6	3757	74	66.81	60	4235090	SAMN29162807	2020	Czech Republic	unknown
GCA_024126695.1	S 246-4	*S. maltophilia*	4420	77	66.21	117	4880609	SAMN29162804	2020	Czech	unknown

										Republic	
GCA_024126755.1	S 248-2	*S. maltophilia*	4181	74	66.55	95	4602555	SAMN29162802	2020	Czech Republic	unknown
GCA_024126795.1	S 252-3	*S. maltophilia*	4357	80	66.38	128	4800142	SAMN29162801	2020	Czech Republic	unknown
GCA_024126805.1	VE 193-12	*S. lactitubi*	4237	68	66.05	66	4672067	SAMN29162800	2020	Czech Republic	unknown
GCA_024127355.1	VE 245-2	*S. maltophilia*	4179	74	66.55	94	4601834	SAMN29162799	2020	Czech Republic	unknown
GCA_024127395.1	VE 245-1	Genospecies 1	4392	74	66.05	85	4838795	SAMN29162798	2020	Czech Republic	unknown
GCA_024127455.1	S 246-1	Genospecies 9	3993	72	66.19	93	4515425	SAMN29162803	2020	Czech Republic	unknown
GCA_024128235.1	S 249-2	*S. maltophilia*	4178	74	66.56	78	4595434	SAMN29162805	2020	Czech Republic	unknown
GCA_024128245.1	VE 244-3	*S. maltophilia*	4180	74	66.55	106	4601540	SAMN29162797	2020	Czech Republic	unknown
GCA_024128295.1	VE 244-1	*S. maltophilia*	4406	77	66.26	135	4853721	SAMN29162796	2020	Czech Republic	unknown
GCA_024128335.1	VA 158-8	*S. sepilia*	4118	74	66.48	72	4584514	SAMN29162795	2020	Czech Republic	unknown
GCA_024128375.1	S 297-3	Genospecies 6	3750	70	66.81	52	4234492	SAMN29162806	2020	Czech Republic	unknown
GCA_024609035.1	pD1b	*S. sepilia*	4202	73	66.45	49	4686110	SAMN30141920	2020	China	BALs
GCA_024734725.1	SCAID WND1-2022	*S. maltophilia*	4429	81	66.17	2	4919031	SAMN30333624	2022	Kazakhstan	swab from purulent wound

	(370)										
GCA_025200825.1	HW002Y	*S. geniculata*	4058	77	66.46	1	4508936	SAMN28910278	2022	Malaysia	hospital ICU ward
GCA_025426215.1	CW002SM	*S. maltophilia*	4527	78	66.12	1	4959065	SAMN30637997	2022	Malaysia	clinical waste
GCA_025617355.1	CGMCC 1.1788	*S. maltophilia*	4531	76	66.19	70	4956694	SAMN30661376	2011	China	unknown
GCA_025642255.1	SG.Y2	Genospecies 6	3647	79	66.94	1	4120518	SAMN30996861	2021	China, Hong Kong	rhizosphere
GCA_026428295.1	CYZ	*S. maltophilia*	4077	79	66.65	1	4517685	SAMN09720998	2017	China	sputum
GCA_027594805.1	454	*S. maltophilia*	4191	78	66.33	1	4666431	SAMN23434718	unknown	Germany	sputum
GCA_027941475.1	SM79	*S. pavanii*	3976	49	67.09	132	4445889	SAMN23458238	2019	Brazil	cloacal swab
GCA_900113425.1	IAM 12423	Genospecies 6	3648	71	66.94	9	4144460	SAMN04487782	unknown	unknown	unknown
GCA_900186865.1	NCTC10257	*S. maltophilia*	4562	80	66.12	1	5004261	SAMEA4076705	1900/1961	unknown	Mouth
GCA_900475405.1	NCTC10258	*S. maltophilia*	3993	79	66.63	1	4481118	SAMEA3856672	1900/1961	unknown	cerebrospinal fluid
GCA_900475685.1	NCTC10498	*S. maltophilia*	4191	78	66.45	1	4661352	SAMEA3956094	1957	unknown	Oropharyngeal swab
GCA_900636655.1	NCTC13014	*S. africana*	4130	80	66.29	1	4592627	SAMEA3724090	1900/2007	unknown	unknown
GCA_900636905.1	NCTC10259	*S. muris*	4110	78	66.84	1	4563878	SAMEA4537264	1900/1961	unknown	unknown
GCA_902156485.2	A1_129041_ seineetmarne	Genospecies 8	3716	72	66.67	83	4277620	SAMEA5749682	unknown	France	unknown
GCA_902386495.1	MGYG-HG UT-02381	Genospecies 8	3598	63	66.81	63	4114905	SAMEA5851885	unknown	unknown	human gut
GCA_902387615.1	MGYG-HG UT-02481	*S. muris*	4448	64	66.61	176	4867831	SAMEA5851986	unknown	unknown	human gut

GCA_918593995.1	S. maltophilia 1800	*S. geniculata*	4432	75	66.19	1	4837108	SAMEA10558844	unknown	Algeria	industrial effluent
GCA_946220905.1	Zyri9ZH5M6_bin.2.MAG	*S. muris*	4228	57	66.63	123	4677595	SAMEA110089041	unknown	USA	human skin
GCA_946220995.1	oRT8oxdYnV_bin.3.MAG	Genospecies 1	4413	66	66.11	122	4845803	SAMEA110089037	unknown	USA	human skin

Note: Strains with light blue shading are Smc strains.

TABLE S3 The MIC (µg/mL) of 15 antibiotics in 90 Smc strains.

Species	Strain	Antibiotic[a]														
		LVX	MH	CAZ	SXT	SAM	TZP	CRO	FEP	ATM	ETP	IPM	AN	GM	NN	CIP
S. geniculata	SMYL1	4 (I)	8 (I)	≥32 (R)	2/38 (S)	≥32	64	≥64	16	≥64	≥8	≥16	32	8	8	≥4
S. sepilia	SMYL10	4 (I)	2 (S)	4 (S)	≤1/19 (S)	≥32	64	≥64	16	≥64	≥8	≥16	32	8	8	≥4
P. hibiscicola	SMYL100	2 (S)	≤1 (S)	4 (S)	≤1/19 (S)	≥32	8	≥64	≥64	≥64	≥8	≥16	≥64	8	8	2
S. maltophilia	SMYL101	≤1 (S)	≤1 (S)	16 (I)	≤1/19 (S)	≥32	8	≥64	≥64	≥64	≥8	≥16	≥64	≥16	≥16	1
S. sepilia	SMYL102	2 (S)	2 (S)	≤1 (S)	≤1/19 (S)	≥32	8	≥64	4	≥64	≥8	≥16	≥64	≥16	≥16	1
S. maltophilia	SMYL103	≤1 (S)	2 (S)	16 (I)	≤1/19 (S)	≥32	8	≥64	16	≥64	≥8	≥16	≥64	≥16	≥16	1
S. maltophilia	SMYL104	≤1 (S)	≤1 (S)	8 (S)	≤1/19 (S)	≥32	8	≥64	≥64	≥64	≥8	≥16	≥64	≥16	≥16	2
P. hibiscicola	SMYL105	≤1 (S)	≤1 (S)	4 (S)	≤1/19 (S)	≥32	8	≥64	2	≥64	≥8	≥16	≥64	8	8	1
S. pavanii	SMYL107	2 (S)	≤1 (S)	4 (S)	≤1/19 (S)	≥32	8	≥64	≥64	≥64	≥8	≥16	≥64	≥16	≥16	2
S. maltophilia	SMYL109	≤1 (S)	≤1 (S)	8 (S)	≤1/19 (S)	≥32	16	≥64	32	≥64	≥8	≥16	≥64	≥16	≥16	2
Genospecies 1	SMYL11	≤1 (S)	≤1 (S)	2 (S)	2/38 (S)	16	8	≥64	4	≥64	≥8	≥16	8	2	2	1
S. maltophilia	SMYL111	≤1 (S)	≤1 (S)	8 (S)	≤1/19 (S)	≥32	8	≥64	≥64	≥64	≥8	≥16	≥64	≥16	≥16	2
P. hibiscicola	SMYL112	≥8 (R)	4 (S)	8 (S)	≤1/19 (S)	16	64	≥64	32	≥64	≥8	≥16	≥64	8	8	≥4
S. maltophilia	SMYL113	≤1 (S)	≤1 (S)	≥32 (R)	≤1/19 (S)	≥32	64	≥64	≥64	≥64	≥8	≥16	≥64	≥16	≥16	2
S. sepilia	SMYL12	≤1 (S)	≤1 (S)	2 (S)	≤1/19 (S)	≥32	64	≥64	16	≥64	≥8	≥16	32	8	8	1
S. muris	SMYL13	≤1 (S)	≤1 (S)	2 (S)	≤1/19 (S)	≥32	8	32	≤1	≥64	≥8	≥16	≥64	≥16	≥16	1
S. maltophilia	SMYL14	≤1 (S)	≤1 (S)	8 (S)	≤1/19 (S)	≥32	8	≥64	≥64	≥64	≥8	≥16	≥64	≥16	≥16	2
S. sepilia	SMYL15	≤1 (S)	≤1 (S)	4 (S)	≤1/19 (S)	≥32	64	≥64	16	≥64	≥8	≥16	32	8	8	2
S. maltophilia	SMYL16	4 (I)	≤1 (S)	8 (S)	≤1/19 (S)	≥32	≥128	≥64	16	≥64	≥8	≥16	≥64	≥16	≥16	≥4
S. geniculata	SMYL19	≥8 (R)	2 (S)	≥32 (R)	2/38 (S)	≥32	≥128	≥64	≥64	≥64	≥8	≥16	32	8	4	≥4
S. maltophilia	SMYL2	2 (S)	4 (S)	≥32 (R)	2/38 (S)	≥32	32	≥64	16	≥64	≥8	≥16	32	8	8	≥4
Genospecies 1	SMYL20	≤1 (S)	≤1 (S)	2 (S)	≤1/19 (S)	≥32	8	≥64	32	≥64	≥8	≥16	4	4	2	2
S. maltophilia	SMYL21	≤1 (S)	≤1 (S)	2 (S)	2/38 (S)	≥32	64	≥64	16	≥64	≥8	≥16	≥64	8	≥16	≤0.5

S. geniculata	SMYL22	≤1 (S)	≤1 (S)	≥32 (R)	≤1/19 (S)	≥32	64	≥64	≥64	≥64	≥8	≥16	16	8	8	≤0.5
S. muris	SMYL23	≥8 (R)	2 (S)	8 (S)	≥4/76 (R)	≥32	32	≥64	16	≥64	≥8	≥16	32	8	4	≥4
S. maltophilia	SMYL25	≤1 (S)	≤1 (S)	4 (S)	≤1/19 (S)	≥32	8	≥64	32	≥64	≥8	≥16	≥64	≥16	8	2
S. maltophilia	SMYL26	2 (S)	≤1 (S)	8 (S)	≤1/19 (S)	≥32	8	≥64	≥64	≥64	≥8	≥16	≥64	≥16	≥16	2
S. sepilia	SMYL27	≤1 (S)	≤1 (S)	2 (S)	2/38 (S)	≥32	≥128	≥64	≤1	≥64	≥8	≥16	≥64	≥16	8	1
Genospecies 7	SMYL28	2 (S)	≤1 (S)	≥32 (R)	≤1/19 (S)	≥32	64	≥64	16	≥64	≥8	≥16	8	2	2	≥4
S. maltophilia	SMYL29	≤1 (S)	≤1 (S)	8 (S)	≤1/19 (S)	≥32	8	≥64	≥64	≥64	≥8	≥16	≥64	≥16	≥16	2
S. maltophilia	SMYL3	≤1 (S)	≤1 (S)	≥32 (R)	≤1/19 (S)	≥32	64	≥64	16	≥64	≥8	≥16	8	4	8	≤0.5
S. maltophilia	SMYL33	≤1 (S)	≤1 (S)	≥32 (R)	≤1/19 (S)	≥32	64	≥64	16	≥64	≥8	≥16	8	4	8	≤0.5
S. maltophilia	SMYL34	≤1 (S)	≤1 (S)	8 (S)	2/38 (S)	≥32	16	≥64	≥64	≥64	≥8	≥16	≥64	≥16	≥16	2
Genospecies 2	SMYL36	2 (S)	≤1 (S)	≥32 (R)	≤1/19 (S)	≥32	16	≥64	≥64	≥64	≥8	≥16	16	8	≥16	≥4
S. muris	SMYL37	≤1 (S)	≤1 (S)	2 (S)	≤1/19 (S)	≥32	≥128	≥64	16	≥64	≥8	≥16	8	8	2	1
S. muris	SMYL38	4 (I)	≤1 (S)	16 (I)	≤1/19 (S)	≥32	≥128	≥64	16	≥64	≥8	≥16	≥64	≥16	≥16	2
S. geniculata	SMYL4	2 (S)	≤1 (S)	≥32 (R)	≤1/19 (S)	≥32	64	≥64	16	≥64	≥8	≥16	32	8	8	≥4
S. maltophilia	SMYL40	≤1 (S)	≤1 (S)	≥32 (R)	≥4/76 (R)	≥32	≥128	≥64	16	≥64	≥8	≥16	≥64	≥16	≥16	1
Genospecies 4	SMYL41	2 (S)	≤1 (S)	4 (S)	≤1/19 (S)	≥32	64	≥64	16	≥64	≥8	≥16	8	8	4	≥4
P. hibiscicola	SMYL42	≤1 (S)	≤1 (S)	4 (S)	≤1/19 (S)	≥32	8	≥64	4	≥64	≥8	≥16	≥64	≥16	≥16	≥4
S. sepilia	SMYL44	2 (S)	≤1 (S)	2 (S)	≤1/19 (S)	≥32	8	≥64	≥64	32	≥8	≥16	≥64	≥16	≥16	2
S. geniculata	SMYL49	2 (S)	≤1 (S)	≥32 (R)	≤1/19 (S)	≥32	16	≥64	≥64	≥64	≥8	≥16	≥64	8	8	2
S. maltophilia	SMYL5	≤1 (S)	≤1 (S)	≥32 (R)	≤1/19 (S)	≥32	64	≥64	16	≥64	≥8	≥16	8	4	8	≤0.5
S. maltophilia	SMYL51	2 (S)	≤1 (S)	8 (S)	≤1/19 (S)	≥32	8	≥64	8	≥64	≥8	≥16	≥64	≥16	≥16	2
S. maltophilia	SMYL52	≤1 (S)	≤1 (S)	8 (S)	≤1/19 (S)	≥32	≤4	32	2	32	≥8	≥16	8	≥16	≥16	≤0.5
S. pavanii	SMYL54	≥8 (R)	2 (S)	2 (S)	≤1/19 (S)	≥32	≥128	≥64	2	≥64	≥8	≥16	≥64	≥16	≥16	2
S. maltophilia	SMYL55	≥8 (R)	8 (I)	≥32 (R)	2/38 (S)	≥32	≥128	≥64	16	≥64	≥8	≥16	8	2	2	≥4
S. sepilia	SMYL56	≤1 (S)	≤1 (S)	4 (S)	≤1/19 (S)	≥32	64	≥64	16	≥64	≥8	≥16	32	8	8	1
S. sepilia	SMYL57	2 (S)	≤1 (S)	8 (S)	≤1/19 (S)	≥32	≥128	≥64	32	≥64	≥8	≥16	≥64	≥16	≥16	2

S. maltophilia	SMYL58	2 (S)	≤1 (S)	≥32 (R)	≤1/19 (S)	≥32	64	≥64	16	≥64	≥8	≥16	32	8	8	≥4
S. maltophilia	SMYL59	2 (S)	2 (S)	≥32 (R)	≤1/19 (S)	≥32	32	≥64	≥64	≥64	≥8	≥16	32	8	≥16	1
S. maltophilia	SMYL6	≤1 (S)	≤1 (S)	≥32 (R)	≤1/19 (S)	≥32	64	≥64	16	≥64	≥8	≥16	8	4	8	≤0.5
S. muris	SMYL60	≤1 (S)	≤1 (S)	2 (S)	≤1/19 (S)	≤2	8	≥64	4	32	≥8	≥16	≥64	≥16	≥16	1
S. geniculata	SMYL61	≤1 (S)	≤1 (S)	≥32 (R)	≤1/19 (S)	≥32	32	≥64	≥64	≥64	≥8	≥16	32	8	8	1
S. geniculata	SMYL62	≤1 (S)	≤1 (S)	≥32 (R)	≤1/19 (S)	≥32	8	≥64	32	≥64	≥8	≥16	≥64	≥16	≥16	1
S. sepilia	SMYL63	≤1 (S)	≤1 (S)	≤1 (S)	≤1/19 (S)	16	8	≥64	≤1	≥64	≥8	≥16	≥64	8	8	≤0.5
S. geniculata	SMYL64	≤1 (S)	≤1 (S)	≥32 (R)	2/38 (S)	≥32	8	≥64	≥64	≥64	≥8	≥16	16	8	4	≤0.5
S. sepilia	SMYL67	≤1 (S)	≤1 (S)	4 (S)	≤1/19 (S)	≥32	16	≥64	≥64	≥64	≥8	≥16	≥64	≥16	≥16	1
S. geniculata	SMYL68	≤1 (S)	2 (S)	≥32 (R)	≤1/19 (S)	≥32	16	≥64	≥64	≥64	≥8	≥16	≥64	≥16	8	2
S. maltophilia	SMYL69	≤1 (S)	≤1 (S)	4 (S)	≤1/19 (S)	≥32	8	≥64	32	≥64	≥8	≥16	32	≥16	≥16	2
Genospecies 1	SMYL7	≤1 (S)	≤1 (S)	16 (I)	≤1/19 (S)	≥32	64	≥64	16	≥64	≥8	≥16	8	4	4	2
S. maltophilia	SMYL70	≤1 (S)	≤1 (S)	8 (S)	≤1/19 (S)	≥32	8	≥64	≥64	≥64	≥8	≥16	≥64	≥16	≥16	1
S. sepilia	SMYL71	≤1 (S)	≤1 (S)	≥32 (R)	≤1/19 (S)	≥32	≥128	≥64	≥64	≥64	≥8	≥16	≥64	≥16	≥16	1
S. geniculata	SMYL73	≤1 (S)	≤1 (S)	≥32 (R)	2/38 (S)	≥32	8	≥64	32	≥64	≥8	≥16	≥64	≥16	≥16	≤0.5
S. maltophilia	SMYL74	≤1 (S)	≤1 (S)	≥32 (R)	≤1/19 (S)	≥32	32	≥64	≥64	≥64	≥8	≥16	≥64	≥16	8	≤0.5
S. maltophilia	SMYL75	≤1 (S)	≤1 (S)	≥32 (R)	2/38 (S)	≥32	32	≥64	≥64	≥64	≥8	≥16	≥64	≥16	8	2
S. geniculata	SMYL76	2 (S)	≤1 (S)	≥32 (R)	≤1/19 (S)	≥32	64	≥64	≥64	≥64	≥8	≥16	≥64	8	8	2
S. sepilia	SMYL78	≤1 (S)	≤1 (S)	2 (S)	≤1/19 (S)	≥32	8	≥64	32	32	≥8	≥16	≥64	≥16	8	1
S. maltophilia	SMYL79	≤1 (S)	≤1 (S)	8 (S)	≤1/19 (S)	≥32	16	≥64	32	≥64	≥8	≥16	≥64	≥16	≥16	2
Genospecies 1	SMYL8	≥8 (R)	8 (I)	16 (I)	2/38 (S)	≥32	64	≥64	16	≥64	≥8	≥16	4	2	≤1	≥4
S. maltophilia	SMYL80	2 (S)	≤1 (S)	16 (I)	2/38 (S)	≥32	16	≥64	≥64	≥64	≥8	≥16	≥64	≥16	8	1
Genospecies 5	SMYL82	≤1 (S)	≤1 (S)	4 (S)	2/38 (S)	≥32	32	≥64	32	≥64	≥8	≥16	≤2	≤1	≤1	≤0.5
S. pavanii	SMYL83	≤1 (S)	2 (S)	2 (S)	2/38 (S)	≥32	8	≥64	≥64	≥64	≥8	≥16	≥64	≥16	≥16	2
S. maltophilia	SMYL84	≤1 (S)	≤1 (S)	8 (S)	2/38 (S)	16	8	≥64	≥64	≥64	≥8	≥16	≥64	≥16	8	1
S. maltophilia	SMYL85	≤1 (S)	≤1 (S)	≥32 (R)	≤1/19 (S)	≥32	≥128	≥64	≥64	≥64	≥8	≥16	≥64	≥16	≥16	2

Genospecies 1	SMYL86	≤1 (S)	≤1 (S)	≤1 (S)	≤1/19 (S)	≤2	8	≥64	≤1	≤1	≥8	≥16	4	2	≤1	≤0.5
S. maltophilia	SMYL87	4 (I)	≤1 (S)	8 (S)	≤1/19 (S)	≥32	64	≥64	16	≥64	≥8	≥16	≥64	≥16	≥16	≥4
S. maltophilia	SMYL88	2 (S)	≤1 (S)	8 (S)	≤1/19 (S)	≥32	8	≥64	≥64	≥64	≥8	≥16	≥64	≥16	≥16	2
Genospecies 3	SMYL89	2 (S)	≤1 (S)	16 (I)	≤1/19 (S)	≥32	8	≥64	32	≥64	≥8	≥16	≥64	8	8	2
S. geniculata	SMYL9	≤1 (S)	≤1 (S)	≥32 (R)	≤1/19 (S)	≥32	32	≥64	≥64	≥64	≥8	≥16	8	4	2	2
S. maltophilia	SMYL90	≤1 (S)	≤1 (S)	4 (S)	≤1/19 (S)	≥32	8	≥64	8	32	≥8	≥16	≥64	≥16	≥16	2
S. maltophilia	SMYL91	≤1 (S)	≤1 (S)	8 (S)	≤1/19 (S)	≥32	8	≥64	≥64	≥64	≥8	≥16	≥64	≥16	≥16	2
S. maltophilia	SMYL92	≤1 (S)	≤1 (S)	≥32 (R)	≤1/19 (S)	≥32	32	≥64	≥64	≥64	≥8	≥16	≥64	≥16	≥16	≤0.5
S. sepilia	SMYL93	≤1 (S)	≤1 (S)	≥32 (R)	≤1/19 (S)	≥32	8	≥64	≥64	≥64	≥8	≥16	≥64	≥16	≥16	1
S. maltophilia	SMYL94	≤1 (S)	≤1 (S)	8 (S)	≤1/19 (S)	≥32	8	≥64	8	≥64	≥8	≥16	≥64	≥16	≥16	1
S. maltophilia	SMYL95	≤1 (S)	≤1 (S)	8 (S)	≤1/19 (S)	≥32	8	≥64	≥64	≥64	≥8	≥16	≥64	≥16	≥16	≤0.5
S. pavanii	SMYL96	2 (S)	≤1 (S)	2 (S)	≤1/19 (S)	≥32	≥128	≥64	≥64	32	≥8	≥16	≥64	≥16	≥16	1
S. geniculata	SMYL97	≤1 (S)	≤1 (S)	≥32 (R)	≤1/19 (S)	≥32	≥128	≥64	≥64	≥64	≥8	≥16	32	8	8	1
S. geniculata	SMYL98	≤1 (S)	2 (S)	≥32 (R)	2/38 (S)	≥32	≥128	≥64	≥64	≥64	≥8	≥16	32	8	8	1
S. africana	SMYL99	≤1 (S)	≤1 (S)	≥32 (R)	≤1/19 (S)	≥32	≥128	≥64	≥64	≥64	≥8	≥16	32	8	≥16	2

[a]Antibiotic: LVX, Levofloxacin; MH, Minocycline; CAZ, Ceftazidime; SXT, Trimethoprim/Sulfamethoxazole; SAM, Ampicillin/Sulbactam; TZP, Piperacillin/Tazobactam; CRO, Ceftriaxone; FEP, Cefepime; ATM, Aztreonam; ETP, Ertapenem; IPM, Imipenem; AN, Amikacin; GM, Gentamicin; NN, Tobramycin; CIP, Ciprofloxacin.
S, susceptible; R, resistance; I, intermediate.
